# Genetic Characterization and Chemical Identification of Moroccan *Cannabis sativa* (L.) Seeds: Extraction, and *In Vitro* and *In Silico* Biological Evaluation

**DOI:** 10.3390/plants13141938

**Published:** 2024-07-15

**Authors:** Amira Metouekel, Fadwa Badrana, Rabie Kachkoul, Mohamed Chebaibi, Mohamed Akhazzane, Abdelfattah El Moussaoui, Nadia Touil, Hamid El Amri, Elmostafa El Fahime, Saïd El Kazzouli, Nabil El Brahmi

**Affiliations:** 1 Euromed Research Center, Euromed Faculty of Pharmacy, School of Engineering in Biomedical and Biotechnology, Euromed University of Fes (UEMF), Meknes Road, Fez 30000, Morocco; amira.metouekel@ueuromed.org; 2 Institute of Genetic Analysis of the Royal Gendarmerie in Rabat (LRAM), Ibn Sina Av., Agdal, Rabat 10040, Morocco; fadwa.badrana@uit.ac.ma (F.B.);; 3 Laboratory of Biochemistry, Faculty of Medicine and Pharmacy, Sidi Mohammed Ben Abdellah University, BP 1893, Km 22, Road of Sidi Harazem, Fez 30000, Morocco; rabie.kachkoul@usmba.ac.ma; 4 Ministry of Health and Social Protection, Higher Institute of Nursing Professions and Health Techniques, Fez 30000, Morocco; mohamed.chebaibi@usmba.ac.ma; 5 Faculty of Sciences Dhar El Mahraz, Sidi Mohamed Ben Abdellah University (USMBA), Fez 30000, Morocco; mohamed.akhazzane@usmba.ac.ma; 6 Plant Biotechnology Team, Faculty of Sciences, Abdelmalek Essaadi University, Tetouan 93002, Morocco; abdelfattah.elmoussaoui@usmba.ac.ma; 7 Cell Culture Unit, Center of Virology, Infectious, and Tropical Diseases, Mohammed V Military Hospital, Rabat 10040, Morocco; 8 National Center for Scientific and Technical Research (CNRST), Angle Avenues des FAR and Allal El Fassi, Hay Ryad, Rabat 10102, Morocco

**Keywords:** *Cannabis sativa*, seeds, genetics, ITS gene, THCAS gene, Cannabisin, antioxidant, NADPH oxidase

## Abstract

This study investigated the molecular, phytochemical, and biological aspects of ten local Moroccan traditional landrace *Cannabis* seeds. Genetic polymorphisms were analyzed using DNA barcode determination, revealing two distinct molecular profiles: “*Cannabis*, species *sativa,* subspecies *indica*” and “*Cannabis*, species *sativa*, subspecies *sativa*”. Furthermore, a new sequence was identified by sequencing of the THCA synthase coding gene. Chemical profiling via HPLC-ESI-FULL-MS and GC-MS-MS of AMSD1 maceration extracts revealed 13 non-volatile chemicals, including 3 inactive cannabinoids and 3 polyphenols, and 24 intriguing volatile compounds, including 7 previously unreported in *Cannabis* seed extracts. Moreover, the *in vitro/in silico* analysis provision of biological activities through their antioxidant power, antimicrobial effect, and cytotoxicity potency, as well as antiviral activity, were realized. These results contribute to a thorough comprehension of Moroccan *Cannabis* seeds, illuminating their molecular, phytochemical, and biological features. Furthermore, they highlight the seeds as a potential source of nutritious components with antioxidant properties, offering valuable insights for future research.

## 1. Introduction

For centuries, *Cannabis sativa* L. was an illegal plant in Morocco. However, the Moroccan government has recently legalized and decriminalized its medical use under the law no. 13–21 on the legal use of *Cannabis* [1]. This has prompted an increased interest among scientists and the pharmaceutical industry. *Cannabis* is a subcosmopolitan dioecious flowering plant belonging to the *Canabaceae* family [2]. The number of species in the genus *Cannabis* has been controversial. Some authors have proposed *Cannabis* as a polytype that includes three species namely the *Cannabis sativa*, *C. Indica*, and *C. Ruderalis* [3]. Based on various studies examining morphology, anatomy, photochemistry, and genetics, it is widely accepted that there is a single species of *Cannabis* known as “*Cannabis sativa Linnaeus*.” This species displays remarkable diversity and substantial variation, and it is additionally divided into two subspecies: *Cannabis sativa* (L.) subsp. *sativa* and *Cannabis sativa* (L.) subsp. *indica*. Both of these subspecies encompass both wild and domesticated varieties [4]. The *Cannabis sativa* var. *sativa* is mostly cultivated for its fiber and seeds, whereas the *Cannabis. sativa* var. *indica* is mainly grown for its high content of cannabinoids. Some of these cannabinoids, such as tetrahydrocannabinol (THC), are known to have psychotropic activities [5].

According to two separate studies realized by Hillig and Clarke, the Moroccan local cultivated variety is considered rare because it has undergone an introgressive hybridization with other varieties of *Cannabis* plants [6,7]. In their study, Clarke and Merlin [8] have reported that the *Cannabis* found in northern region of Moroccan is a hybrid between European varieties NLH (narrow-leaf-hemp) or hemp biotype and the pure original Moroccan NLD (Narrow-Leaf-Drug) landrace variety or Moroccan drug-type *Cannabis* [8].

*Cannabis* is currently recognized for its medicinal properties. Since the Neolithic era, hemp has been cultivated for its fibers used in textiles, and its seeds, which are used for food [9]. The *Cannabis* seed is particularly intriguing in the field of nutrition because of its unique ω-6/ω-3 fatty acid ratio of 3:1. This ratio offers various benefits for cardiovascular health, skin, hormonal balance, and insulin resistance [10]. *Cannabis* seeds contain not only essential fatty acids, but also other beneficial natural compounds such as β-sitosterol, which contributes to lower cholesterol levels. Additionally, *Cannabis* contain tocopherols, which have both antioxidant and anticancer activities. These seeds also have sufficient and effective amounts of terpenes, cannabinoids, and certain phenolics, including methyl salicylate, which offers various health benefits [11,12].

The seeds of *Cannabis sativa* possess a diverse range of properties, making them highly promising for the development of various therapeutic, cosmetic, functional foods, and industrial nutraceuticals products. The seeds of Moroccan *Cannabis sativa* L. exhibit diverse genetic and chemical characteristics. They are also rich in nutrients and contain various bioactive compounds including antioxidants, antimicrobials, and probably cytotoxic and antiviral agents. The primary objective of this study is to conduct a thorough investigation and analysis of the genetic and chemical variations present in a set of *Cannabis* seeds from the northern Morocco. This will be achieved by employing a combination of the high-performance liquid chromatography-electrospray ionization-full scan mass spectrometry combined with the gas chromatography–tandem mass spectrometry (TQ) chemical profiling and genomic fingerprinting methodologies. Through the utilization of *in vitro* and *in silico* analyses, our primary aim is to elucidate the functional characteristics of these compounds as bioactive constituents in para-pharmaceutical and nutraceutical formulations. Consequently, the findings not only contribute to the progression of our understanding regarding the genomic and phytochemical resources of Moroccan *Cannabis*, but also provide substantial prospects for future breeding endeavors and the discovery of new bioactive compounds with distinct properties. 

## 2. Material and Methods

### 2.1. Reagents and Materials

All standards, reagents, and solvents used in this study were purchased from Sigma-Aldrich (Saint-Quentin-Fallavier Cedex, France) and were of analytical grade. The biomolecular reagents and biological standards and references were obtained from AMRESCO (Rosny-sous-Bois, France).

### 2.2. Plant Material

**Figure 1:**
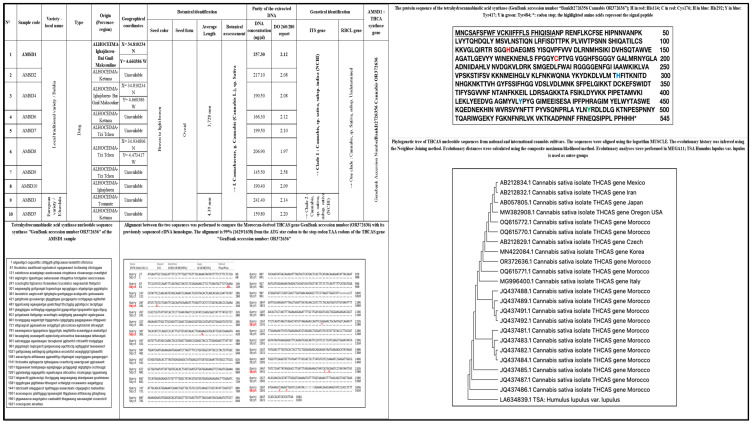
Result of the botanical and genetical identification of the studied plant (seeds) samples: The informed variety is the one described by the cultivars; DNA: Deoxyribonucleic acid; OD: optical density; NCBI: National Center for Biotechnology Information; ITS gene: Internal Transcribed Spacer gene; RBCL gene: Rubisco large subunit gene; THCAS: tetrahydrocannabinolic acid (THCA) synthase; Phylogenetic tree of THCAS nucleotide sequences, Neighbor-Joining method [13]; Composite maximum likelihood method [14]; Evolutionary analyses in MEGA11 [15,16].

The *Cannabis* seed samples were collected from five different regions in the northern of province of Al-Hoceima, Morocco. The collection took place between June and September 2022, after the grain had matured. The data of all the coordinates are listed in Figure 1. A total of ten *Cannabis* seeds were evaluated. The obtained seeds were cleaned, if needed, dusted, crushed, sieved, lyophilized, and subsequently stored at 4 °C, until further use. 

### 2.3. Identification of Plant Material

#### 2.3.1. Botanical Identification

During the harvesting stage, the samples were collected from the farmers. The plant that had been collected was meticulously positioned on Kraft paper, subjected to compression, and subsequently dispatched to the Scientific Institute of Mohammed V University of Rabat, Morocco for the purpose of botanical identification by proficient botanists. Subsequently, the plant specimen was subjected to scanning using the HerbScan system, employing the Epson Expression 10,000 XL flatbed scanner renowned for its exceptional resolution capabilities of 600 DPI. The scanning procedure was carried out using two standardized dimensions. Ultimately, the botanical specimen underwent a process of thermal drying within a specifically designed apparatus, which serves the purpose of eliminating any potential impurities before it is stored.

#### 2.3.2. Genetic Identification

##### DNA Extraction, Quantification, and Amplification

The genetic identification was conducted in order to discern and differentiate the gathered seed samples. Initially, a quantity of 100 mg of *Cannabis* seeds samples was subjected to grinding using the Tissulyser II. Subsequently, the extraction of DNA was performed using the ISOLATE II DNA Plant kit in accordance with the guidelines provided by the manufacturer. In addition, the quantification of DNA was performed using the NanoDrop 8000 spectrophotometer manufactured by ThermoFisher (Waltham, MA, USA). Additionally, a combination of primers a/f, c/e, c/h, d/h, d/b, and g/f, as described by Kojoma et al. (2006) [17], was also employed. The process of sequencing was conducted through the amplification of various fragments of the THCA synthase gene, with the objective of assembling the complete coding region of the THCA synthase gene. The amplification of the THCA synthase gene was performed by combining 1 µL of DNA extract (100 ng/µL) with 14.55 µL of sterile distilled water, 2.5 µL of 10× PCR buffer (Invitrogen, Waltham, MA, USA), 1.25 µL of MgCl_2_ (50 mM), 2 µL of dNTP (10 mM), 1 µL of each primer (10 µM), 2.5 µL of Q-Solution (Qiagen, Manchester, UK), and 0.2 µL of Platinum DNA polymerase (5 U/µL, Invitrogen) in a thermocycler microtube. The PCR amplification procedure was executed in accordance with the established standard protocol. The initial denaturation step involves a preheating process at a temperature of 94 °C for a period of 5 min. This is followed by a series of 35 cycles, each consisting of denaturation at 94 °C for 1 min, annealing at 55 °C for 1 min, and extension at 72 °C for 2 min. The process concludes with a final extension step at 72 °C for 10 min. The experimental procedures were carried out utilizing a BIO RAD S1000 thermal cycler (Hercules, CA, USA).

Subsequently, the amplification of the RBCL and ITS genes was conducted in accordance with the prescribed protocol. A volume of 1 µL of the purified DNA was introduced into a reaction mixture with a total volume of 25 μL. The reaction mixture comprised 15.5 µL of water, 5 µL of buffer (MyTaq, manufactured by Bioline in the United States, London, UK), 0.25 µL of reverse primer, 0.25 µL of forward primer, 0.5 µL of Taq enzyme, and 0.5 µL of deoxyribonucleotide triphosphates (dNTPs). The polymerase chain reaction (PCR) amplification was conducted using a Biometra-T-advanced thermal cycler, following the specified parameters: The experimental procedure consisted of several steps: (a) an initial denaturation stage at 95 °C for 120 s; (b) subsequent denaturation at 95 °C for 30 s; (c) a series of 35 hybridization cycles at a temperature of 55 °C for 30 s each; (d) annealing at 72 °C for 30 s; and finally, (e) a final annealing stage at 72 °C for 30 s.

The rbcl and ITS universal primers, which have been previously referenced, were chosen to amplify the barcode regions. This selection was made in order to assess genetic variation and facilitate the identification and classification of the various *Cannabis* seed samples available to us. In order to conduct genetic identification, a singular sample was subjected to the utilization of *Cannabis*-specific primers targeting THCA synthase.

##### Sequencing and Data Analysis

The amplified products underwent purification through the ExoSAP-IT PCR cleanup Kit, manufactured by Applied Biosystems (Foster City, CA, USA). Following this, sequence reactions were executed using the Big Dye terminator cycle sequencing kit version 3.1 from Applied Biosystems. The recommended thermal cycling parameters for the cycle sequencing reaction were as follows: an initial denaturation at 96 °C for 60 s, succeeded by 25 cycles of denaturation at 96 °C for 10 s, annealing at 50 °C for 5 s, and extension at 60 °C for 240 s, followed by an infinite hold at 4 °C. The purification of sequencing products was conducted using BigDye^®^ XTerminator™ Purification Kit (Applied Biosystems) and subsequently loaded into an ABI 3130xL capillary sequencer (Applied Biosystems) in accordance with instructions provided by the manufacturer. The sequencing data obtained from the two markers for each sample were analyzed using the software Unipro Ugene (version 47.0) and BioEdit (version 7.1.9) following capillary electrophoresis. The phylogenetic analysis was performed using the software MEGA11 (version 11.0.13) (https://www.megasoftware.net/, accessed on 13 June 2023) using Maximum Parsimony method. Bootstrap support (BS) values for individual clades were calculated by running 1000 bootstrap replicates of the data.

### 2.4. Nutritional Quality of Cannabis Seeds

The elemental nutritional composition of *Cannabis* seeds was determined by Inductively Coupled Plasma–Atomic Emission Spectrometry (ICP-AES, Horiba Jobin Yvon, Piscataway, NJ, USA), with argon plasma. The calibration of the instrument is performed using standard solutions prepared from Merck’s high-quality Certipur^®^ standard solutions, single and multi-element. The analysis parameters were fixed at 1200 W for power, 12 L/min as a volume of plasma gas, 0.2 L/min for cladding gas, and without auxiliary gas. For sample preparation, 0.2 g of pretreated seeds was dissolved with nitric acid and hydrogen peroxide for 18 h. The mixture was processed under controlled pressure and temperature for 35 min. The reaction mixture was filtered through a 0.22-micron filter (Millex^®^-GP (Rahway, NJ, USA), 0.22 µm, Filter Unit), and then diluted before the analysis. A blank was prepared in the same conditions. The H_2_O value was established by calculating the dry weight before and after the lyophilization process at 0.5 mBar pressure for one night. The fat content was determined by Soxhlet extraction with petroleum ether organic solvent, according to AOAC 920.39 [18]. Total mineral percent was determined as ash by incineration at 550 ± 15 °C, according to AOAC 923.03 [18,19]. 

### 2.5. Phytochemical Analysis of Cannabis Seeds Extracts

The extraction procedure included a 24 h dynamic maceration at room temperature with seven different organic solvents arranged in ascending order of polarity (hexane, ethyl ether, chloroform, acetone, ethanol, methanol, and distilled water in a 1:7 ratio, respectively). The procedure was carried out with the aid of controlled magnetic stirring. Concentration of the individual extracts was accomplished using a rotary evaporator set at 30 °C. They were then refrigerated at a temperature of −4 °C to permit subsequent investigation.

#### 2.5.1. Colorimetric Dosage

##### Total Polyphenol Content (TPC)

Total polyphenol content was determined by the Folin–Ciocalteu colorimetric method as described by Singleton et al. [20] with slight modifications. Briefly, 1 mL of each extract was mixed with 500 µL of Folin–Ciocalteu reagent and incubated at room temperature for 8 min. Then, 2 mL of Na_2_CO_3_ solution (7.5%) was added. Using an UV-Visible JENWAY 7305 spectrometer (Vernon Hills, IL, USA) the absorbance was measured at 765 nm after incubation of 30 min in the dark. Finally, the results are expressed as milliequivalents of gallic acid per gram of the extract (mg EGA/g).

##### Total Flavonoid Content (TFC)

The flavonoid content was assessed using a UV-Visible JENWAY 7305 spectrometer [21]. Each extract (0.5 mL) was mixed with aluminum chloride (0.1 mL, 10%), potassium acetate (0.1 mL, 1 M), methanol (1.5 mL, 80%), and distilled water (2.8 mL). After a 30 min incubation at room temperature, absorbance was measured at 415 nm. A blank was prepared under the same conditions, and Quercetin was served as the standard. Flavonoid concentration is expressed as milliequivalents of quercetin (EQ) per gram of extract (mg QE/g).

##### Total Flavones Content (TF)

Flavones determination was conducted using the aluminum chloride test’s colorimetric method [22]. For each sample, 1 mL was combined with 1 mL of aluminum chloride (2%) and 3 mL of sodium acetate (5%). After centrifugation at 3000 rpm for 20 min to obtain a clear solution, absorbance was measured at 440 nm. An internal calibration curve was established using Quercetin ranging from 0 to 100 μg/mL, and the results are reported as milligrams of quercetin equivalent (QE) per gram of extract (mg QE/g).

##### Total Anthocyanin Content (TAC)

The total anthocyanin content was assessed using the differential pH method involving the measurement of absorbance at pH values of 1.0 and 4.5 [23]. Specifically, 0.5 mL of the extract was introduced to 3.5 mL of potassium chloride buffer (0.025 M) to attain a pH of 1.0. The mixture was stirred and incubated for 15 min, then measured by spectrophotometer at 515 and 700 nm. For the second pH (4.5), the extract was also mixed with sodium acetate buffer (0.025 M) in the same way, and then the absorbance was measured at the same wavelengths after a period of 15 min of incubation. The results are expressed as milligrams of cyanidin-3-glucoside equivalents per gram of extract and calculated according to the following standard equation:

$$TA=\frac{A\times MW\times DF\times 10^{3}}{\varepsilon \times L}$$

TA = total anthocyanin, *A* (absorbance) = ((A515 − A700) pH = 1 − (A515 − A700) pH = 4.5); *MW* = molar weight of cyanidin 3-glucoside (449.2 g/mol); *DF*: dilution factor; *ε*: extinction coefficient (L·cm^−1^·mol^−1^) = 26,900 for cyanidin 3-glucoside; *L*: optical path (cm); $10^{3}$: conversion factor from g to mg.

#### 2.5.2. Chromatographic Analysis

##### HPLC-ESI-FULL-MS Analysis

The analysis of the different seed extracts was conducted by HPLC-ESI-FULL-MS. Before injection, the samples underwent filtration using a disposable LC syringe filter disc with a pore size of 0.45 µm (Millex^®^-GP, 0.45 µm). The chromatography technology used is the DIONEX UltiMate 3000 HPLC Extractive Plus System (UHPLC^+^ by ThermoFisher Scientific) equipped with a diode array detector (DAD) coupled to an electrospray ionization mass spectrometer (ESI-MS/MS) detector with high resolution quadrupole—Orbitrap™ and a mass range: 50–6000 u*m*/*z*. The chromatograms were processed using the Thermo Scientific Chromeleon Chromatography Data System (CDS) (Thermo Xcalibur Roadmap version 4.1). Chromatographic separation was performed with a Thermo Scientific™, Hypersil™ BDS C18 (150 mm, 4 mm × 6 mm, 5 μm) column. Mass detection was performed in negative mode, using a Thermo Scientific™, Exactive™ Plus Orbitrap Mass Spectrometer equipped with an Electrospray Ionization (ESI) source. The mobile phases consisted of (A) acidified water with citric acid (adjusted to an appropriate pH of 3.1) and (B) MeOH. The analysis was conducted in gradient mode, employing different pillars with suitable polarity for 45 min. The molecules detected in each extract chromatogram were screened against the Nist MS Search 2.3 MS-library and MSMS-library databases. This method was designed specifically for *Cannabis* seed extracts, and it is still undergoing optimization and validation. 

##### Specific GC-MS-MS (TQ) Analysis

To convert the fixed compounds in the extracts into low-molecular-weight volatile compounds suitable for gas chromatography analysis, a trimethylsilylation derivatization was performed on all seven extracts. An aliquot of the extract (equivalent to one eq.) was evaporated and then silylated using *N*-trimethylsilyl-*N*-methyl trifluoroacetamide (MSTFA). Subsequently, 0.5 μL of the resultant mixture was introduced into a gas chromatograph equipped with a mass spectrometer and a triple quadrupole detector (Shimadzu-GCMS-TQ8040 NX Triple Quad Gas Chromatograph Mass Spectrometer (Tokyo, Japan). The chromatographic separation was carried out on an Apolar capillary RTxi-5 Sil MS column (30 m × 0.25 mm ID × 0.25 µm). Helium was used as the carrier gas. The injection volume was 1 µL in splitless mode, and the analysis time was set to 50 min. The ion source temperature was maintained at 200 °C, the interface temperature at 280 °C, and the injection temperature at 250 °C. The pressure was set to 37.1 kPa. The temperature program began at 50 °C for 2 min, followed by an increase of 5 °C/min to 160 °C for 28 min, and finally, the temperature was increased using the same gradient to reach 280 °C for 20 min. This method was specifically developed to detect and quantify even trace amounts of substances.

### 2.6. Biological Activities

#### 2.6.1. Antioxidant Effect

The antioxidant capacity of the seven extracts was evaluated *in vitro* using three assays. First, the DPPH method consists of adding 100 µL of each extract solution to 750 µL of a methanolic solution of DPPH (0.004%) [24]. Absorbance was assessed at 517 nm against a blank after a 30 min incubation at room temperature. Methanol replaced the sample for the negative control. The experiments were conducted in triplicate, and the results are presented as the percentage of DPPH inhibition, calculated using the following equation:

$$PI\left(\%\right)=\frac{A0-A}{A0}\times 100$$

*PI*: Percentage of inhibition; *A*0: Absorbance of the DPPH of negative control (NC); *A*: Absorbance of DPPH of sample. The IC_50_ was calculated from the graph plotted of inhibition percentage against extract concentration. 

The reducing power test (FRAP) [24] was performed by adding 500 µL of phosphate buffer (0.2 M, pH = 6.6) to 500 µL of potassium ferricyanide (1%) and 100 µL of different concentrations of the samples dissolved in methanol. After 20 min of incubation at 50 °C, 500 µL of aqueous TCA solution (10%), 500 µL of distilled water, and 100 µL of 0.1% FeCl_3_ were added to the reaction medium. The absorbance was determined at 700 nm against the blank. The results are expressed as 50% effective concentration (EC_50_) reflecting the concentration of antioxidant required to obtain an absorbance of 0.5 nm.

The total antioxidant capacity (TAC) [25] test was performed by mixing 25 µL of the extract with 1 mL of reagent solution (0.6 M sulfuric acid, 28 mM sodium phosphate, and 4 mM ammonium molybdate) and then incubating at 95 °C for 90 min. The absorbance was measured by spectrophotometer at 695 nm using a blank containing 25 µL of methanol instead of the extract. The total antioxidant capacity was expressed as micrograms of ascorbic acid equivalent per gram of extract (µg EAA/g extract) from an ascorbic acid standard curve.

#### 2.6.2. Antimicrobial Activity

The antibacterial activity was first evaluated qualitatively by the disc diffusion method [26] to screen and select only the active extracts. All samples were tested at different concentrations ranging from 5 to 100 mg/mL against *Escherichia coli* (ATCC B703), *Pseudomonas aeruginosa* (ATCC B612), *Bacillus cereus* (ATCC B1167), *Enterococcus faecalis* (ATCC B392), *Staphylococcus aureus* (ATCC B803), *Listeria monocytogenes* (ATCC B806). Then, the extracts judged active underwent microdilutions on 96-well microplate to study quantitatively and follow their activity kinetics using a spectrophotometer (BioTek, Epoch™ 2 Microplate Spectrophotometer, Santa Clara, CA, USA). The active extracts were studied by the microdilution method (at concentration from 0.039 to 10 mg/mL) and the kinetics of their antibacterial activity against the same microorganisms was followed by applying the following spectrophotometer program with a shake before each read: read speed: normal; kinetic time: 24 h; kinetic interval: 1 h; wavelength: 600 nm; set point: 37 °C. In addition, and for a complementary purpose, the resazurin test was also realized. The positive controls used in this test are ampicillin and penicillin. The tests were carried out in triplicate. For the antifungal activity, the same process was used against *Candida albicans* (IHEM 15856), *Aspergillus niger* (IHEM 16879), and *Penicillium crustosum* (MUCL 41820).

#### 2.6.3. Cytotoxicity

Monkey kidney cells (Vero) were cultured in Dulbecco’s modified Eagle’s medium (DMEM) (Sigma-Aldrich, Grand Island, NE, USA) supplemented with 10% fetal calf serum, L-glutamine (2 mmol/L). To perform bioassays, cells were seeded in 96-well plates at a concentration of 3 × 10^5^ viable cells/mL (3 × 10^4^ cells/well). Trypan blue staining (Sigma-Aldrich, St. Louis, MO, USA) was used to determine cell viability. To assess cytotoxicity on uninfected Vero cells, dilutions of the extracts ranging from 200 µg/mL to 1.5 µg/mL were used. After 72 h of incubation, cytotoxicity was determined by microscopic examination of the cell morphology. The concentration at which the number of cells was reduced to 50% of that of the controls was considered the 50% cytotoxic concentration (CC_50_). All tests were performed in triplicate [27].

#### 2.6.4. Antiviral Activity

The Wuhan+614G and the Delta variants of SARS-CoV-2 virus were used to study the antiviral activity of *Cannabis* seeds extracts. Different concentrations ranging from 200 µg/mL to 1.5 µg/mL were prepared in the maintenance medium and added to Vero cells grown in 1-day-old 96-well plates just before inoculation at a multiplicity of infection (MOI) of 0.01 TCID50 per cell. The plates were incubated at 37 °C with 5% CO_2_ for 72 h. The examination of cell toxicity and the appearance of cytopathic effect (CPE) were performed every 24 h [28].

### 2.7. Molecular Docking

The 2D configuration of all molecules identified in *Cannabis sativa* L. seeds by GC-MS-MS(TQ) or HPLC-ESI-FULL-MS was obtained from the PubChem online database. Subsequently, Schrödinger Maestro’s LigPrep wizard was utilized to convert these compounds into their respective minimized 3D structures. Epik was employed to predict the potential ionization states at a target pH range of 7.0 ± 2.0. To ensure the preservation of specified chiralities, up to the maximum of 32 stereoisomers per ligand were produced. The force field OPLS3 was employed in these computations [29].

The target protein utilized for molecular docking was obtained from the RCSB Protein Data Bank. For assessing antioxidant activity, NAD(P)H Oxidase was chosen (PDB id: 2CDU) [30]. Additionally, the following proteins were employed to evaluate antimicrobial activity: beta-ketoacyl-[acyl carrier protein] synthase from *E. coli* (PDB id: 1FJ4) [31], Staph aureus nucleoside diphosphate kinase (PDB id: 3Q8U) [31], a beta-1,4-endoglucanase from Aspergillus niger (PDB id: 5I77) [32], and sterol 14-alpha demethylase (CYP51) from the pathogenic yeast *Candida albicans* (PDB id: 5FSA) [33].

The 3D structure of the protein was prepared using the protein preparation wizard available in Schrödinger Maestro (v11.1). During this process, various steps were carried out, including the assignment of bond orders, utilizing the CCD database, addition of hydrogen atoms, creation of zero-order bonds for metals and disulfide bonds, conversion of selenomethionines to regular methionines, and removal of water molecules beyond 5 Å from het groups. The default pH of 7.0 ± 2.0 was maintained using Epik for handling the het state. Finally, the restrained minimization was performed under the OPLS3 force field, leading to the convergence of heavy atoms to an RMSD of 0.30 Å [29].

For ligand docking, a specific atom within the ligand was selected, and a default grid box was generated with a volumetric spacing of 20 × 20 × 20. Subsequently, the ligand was docked onto the protein’s grid box using the ‘Extra Precision’ (XP) method. To evaluate the docking results, the XP GScore was used. The docking of ligands with flexibility was executed utilizing the Glide module within Schrödinger-Maestro (v11.5) employing standard precision (SP). To enhance the accuracy, penalties were enforced for non-cis/trans amide bonds, and specific parameters for ligand atoms were set, including a Van Der Waals scaling factor of 0.80 and a partial charge cutoff of 0.15. The scoring of docking outcomes involved considering energy-minimized poses, and the evaluation relied on the Glide score. The top-docked pose, characterized by the lowest Glide score value for each ligand, was identified and recorded for subsequent analysis [33].

### 2.8. Statistical Analysis

The outcomes of experiments conducted in triplicate were presented as the mean ± standard deviation (SD). The raw data underwent processing using Microsoft Excel and GraphPad Prism Windows 7.05 software. Statistical analyses were performed using GraphPad Prism version 7.0. Normality and homogeneity were verified using the Shapiro–Wilk and Levene’s tests, respectively. For multiple comparisons as a post hoc test, Tukey’s HSD test was employed. Statistical significance was acknowledged when *p* was less than 0.05.

## 3. Results and Discussion

### 3.1. Plant Identification

The botanical assessment primarily involved the examination of the morphological attributes of each component of the plant, followed by a comparative analysis with reference species. The ten samples that were examined have been classified as *Cannabis sativa*. The plant derived from the AMSD1 sample seeds was recorded and assigned the voucher code “RAB113319” (see attached Supplementary Information). Nevertheless, it seems that relying exclusively on botanical identification may be insufficient for establishing significant differentiation. This led us to conduct a more comprehensive genetic study. 

The extracted genomic DNA from all samples was found to be pure according to the values of ration OD260/280 (1.8 < OD 260/280 < 2) (Figure 1). The ITS and RBCL primers successfully generated clear bands of 500 bp and 750 bp, respectively. These two genes, ITS and RBCL, can serve as barcodes for identifying and authenticating *Cannabis* plant species. The ITS gene facilitated the identification of subspecies and the differentiation between varieties. Similar results were reported by Mello et al. [34] and further confirmed by Anabalón et al. [35]. Moreover, the ITS gene exhibited higher discriminatory power compared to the RBCL gene, making it more suitable for *Cannabis* identification. Based on the obtained results, the studied samples were classified into two major groups. The first group, consisting of eight samples (AMSD1, AMSD2, AMSD4, AMSD6, AMSD7, AMSD8, AMSD9, and AMSD10), displayed a genetic profile closely related to “*Cannabis*, sp. *sativa*, subsp. *indica*.” The second group, including AMSD3 and AMSD5, exhibited a genetic fingerprint associated with “*Cannabis*, sp. *sativa*, subsp. *sativa*.”. Therefore, this molecular study supports the hypotheses proposed by Clarke and Merlin [8], and Bachir et al. [36]. A phylogenetic tree (Figure 1) was constructed using the “MEGA-11” software, employing the neighbor-joining method. The statistical significance of the tree was tested and evaluated through bootstrap analysis to measure the robustness and confidence level of the phylogenetic tree obtained. 

The sequencing of THCA synthase gene sequencing from AMSD1 sample led to the determination of a new original Moroccan sequence of this gene. The sequence was deposit in the GenBank database under accession number “*BankIt2726356 Cannabis OR372636*” (submission ID: 2726356). The results obtained exhibited distinct sequences that were determined to be comparable to universally recognized references for *Cannabis* of the NLD drug type (Figure 1).

The signal peptide of the THCAS gene comprises 28 amino acids [37]. The Moroccan THCAS gene, with a length of 1638 nucleotides and GenBank accession number OR372636, underwent sequencing. Aligning this gene sequence (GenBank accession number: OR372636) with its mRNA precursor (GenBank accession number: AB057805) reveals that the THCAS gene from the Moroccan *Cannabis* variety features an open reading frame spanning 1638 nucleotides, encoding a polypeptide of 545 amino acids. The structure of THCA synthase is identified as a monomeric enzyme with two domains (I and II) separated by FAD, along with two subdomains (Ia and Ib). Specifically, subdomain Ia extends from position 28 to 134 aa, subdomain Ib covers positions 135 to 253 aa and 476 to 545 aa, while domain II spans positions 254 to 475 aa [38].

The anticipated progression of the oxidative cyclization reaction involves an intermediate stage characterized by the removal of hydrogen and a proton from the C3 and O6’ positions of cannabigerolic acid (CBGA). This process initiates the formation of two novel chemical bonds between C3 and C4, as well as between O6’ and C8 in Δ9-tetrahydrocannabinolic acid (THCA). The catalyzed reaction comprises the transfer of a hydrogen from the C3 position of CBGA by flavin adenine dinucleotide (FAD). Concurrently, Tyr484 of the THCA synthase removes a proton from the hydroxyl group at the O6’ position of CBGA. This facilitates the approach of C4 and C8 of the monoterpene moiety of CBGA to C3 and O6’, respectively, culminating in the formation of the new THCA cycle. The proposed catalytic mechanism for the oxidation of CBGA to THCA involves the generation of hydrogen peroxide (H_2_O_2_) to regenerate FAD during catalysis [38]. The crystal structure of THCA synthase (THCAS) reveals critical features, such as the covalent binding of FAD (linked to His114 and Cys176), residues His292 and Tyr417 interacting with the CBGA substrate, disulfide bonding (between Cys37 and Cys99), and the presence of six N-glycosylation sites [39].

The THCAS enzyme protein exhibits specific interactions with the CBGA substrate or the FAD cofactor at crucial positions spanning its entire length. These pivotal amino acid sites were explored in the protein sequence aligned with the THCAS GenBank accession number OR372636. The Moroccan variety’s THCAS gene encodes a polypeptide of 545 amino acids, with the initial 28 amino acids constituting the signal peptide. The predicted mature THCAS polypeptide is 517 amino acids long, with a notable difference in the deduced NH_2_-terminal. Amino acids forming covalent bonds with THCAS (His114 and Cys176) are highlighted in red, those involved in CBGA substrate binding (His292 and Tyr417) in blue, and the catalytic site of the enzyme THCAS (Tyr484) in green. These sites were identified at the same locations as described earlier. The NH_2_-terminal sequence identified as NPRENFLKXFSKHIPNNVANPKLV [40] corresponds to the nucleotide sequence 85–156 Ns, with only two nucleotide mismatches. Consequently, the NH_2_-terminal (GenBank accession number “BankIt2726356 *Cannabis* OR372636”) is NPRENFLKCFSEHIPNNVANPKLV, exhibiting two discordant amino acids. 

In the present phylogenetic study of THCAS nucleotide sequences, the Moroccan varieties GenBank accession numbers (OR372636.1; OQ615770.1; OQ615771.1; OQ615772.1; JQ437481.1; JQ437482.1; JQ437483.1; JQ437484.1; JQ437485.1; JQ437486.1; JQ437487.1; JQ437488.1; JQ437489.1; JQ437491.1; JQ437492.1) were separated from Exotic, American, European, and Asian varieties GenBank accession number (MW382908.1; AB057805.1; MG996400.1; MN422084.1; AB212829.1; AB212834.1; AB212832.1). The OR372636.1 GenBank accession number represents the nucleotide sequence of the THCAS gene of the AMSD1 sample of this study. The OQ615770.1, OQ615771.1, and OQ615772.1 GenBank accession numbers represent the nucleotide sequences of the THCAS gene of three drug-like *Cannabis* plants collected from the Taounate city of the Fez region analyzed, studied, and sequenced by our research team. The JQ437481.1, JQ437482.1, JQ437483.1, JQ437484.1, JQ437485.1, JQ437486.1, JQ437487.1, JQ437488.1, JQ437489.1, JQ437491.1, and JQ437492.1 GenBank accession numbers represent eleven samples of *Cannabis* resin drug type analyzed previously [41]. This remoteness of THCAS nucleotide sequences in the Phylogenetic tree is explained by the presence of multiple mutations in the THCAS gene. *Humulus lupulus* (Common Houblon) is a plant species belonging to the family Cannabaceae native to Europe, Asia, and North America in their temperate region. The analysis of the proximity of this plant (GenBank accession number: LA634839.1) classifies an outer-group due to the phenotypic and genetic differences between the species of the same family.

However, we observed that cultivars in Morocco are not homogeneous populations and that they are organized into lineages that are closely related to each other and are close to or away from other varieties of the other paid varieties. As there is a radical difference in the nucleotide sequence between exotic Moroccan, American, European, and Asian accessions, this explains the mixing and genetic diversity experienced by the Moroccan varieties. Therefore, THCA synthase sequencing can be used to identify if the seized *Cannabis* sample is a Moroccan origin. A previous study on the analysis of the sequence of THCA synthase by Kojoma showed that the types of exotic drugs showed little or no variation indicating that the analysis of the sequence of the THCA synthase can distinguish the accessions of Morocco from those of other countries [17]. Moreover, the distribution of Moroccan varieties of the Moroccan drug type placed in the tree in last position compared to other accessions is consistent with the hypothesis that *Cannabis* was newly introduced by the Arab invasions and subsequently by the European colonization, and subsequently by the introduction of foreign varieties of traffickers from Pakistan and Afghanistan, for use as donors in hybridization and selection, to obtain cultivars adapted to Moroccan climatic and geographical conditions with a high level of THC [39,41] while keeping the originality of Moroccan *Cannabis* offered by its climate and geographical position that give Morocco its first position as a producer of *Cannabis* in the world [42]. 

It is necessary to develop a database of sequences of *Cannabis* plants of different geographical origins to facilitate the analysis and identification of varieties specific to Morocco seized from trafficking or used for biomedical applications. This database will help national and international agencies identify the geographical origin and route of seizures used by smugglers as well as the types of varieties used by Moroccan growers and biomedical associations. The individualization of the native variety, characterized by a THC/CBD ration, a spectrum of cannabinoids, terpenes, and alkaloids homogeneous adapted to biomedical applications, is difficult. Since *Cannabis* is an anemophilic plant, the development of a database can help to recognize this variety, if it still exists, and/or the nearest varieties.

### 3.2. Elemental Nutritional Composition

Hemp seed is now a foodstuff under the EU Regulation on nutrition and health claims made on foods [43]. Its proximate nutritional quality including its energy value was elucidated by several recent works [11,19,44]. In order to evaluate the nutritional composition in terms of essential minerals contained in our AMSD1 *Cannabis* seed, an elemental profiles using ICP-AES was carried out (Table 1). The selection of an element menu was based on the major and minor constituents in the seed materials analyzed. Eleven elements (i.e., Ca, Cr, Cu, Fe, K, Mg, Mn, Mo, Na, P, and Zn) were detected across the AMSD1 sample at levels of mg/kg dry matter, in close agreement with levels previously reported by Menezes et al. [45] for a Romanian variety of *Cannabis* seeds. The elemental analysis of our sample revealed high contents of Ca (573.408 mg/kg), K (3755.999 mg/Kg), Mg (727.008 mg/Kg), Na (309.504 mg/Kg), and P (2085.695 mg/Kg), while Fe, Mn, and Zn were as low as 25.328, 26.974, and 47.615 mg/Kg, respectively. However, Cu and Cr were not detected. As shown, the levels of the intake of the different inorganic elements are generally lower in AMSD1 seeds, compared to the recommended daily intake conformed by FDA. The studied seeds also represent a comparable mineral composition with those of the Romanian variety of a *Cannabis* seeds studied by Menezes et al. [45]. Therefore, these results confirm the good nutritional quality of the studied Moroccan *Cannabis* seed but at moderately lower mineral quantities than the compared European varieties. Concerning the rate of H_2_O is about 3.85%, the fat content is 30.25%, and the total mineral percentage is 6.97%. These values agree with those data reported to date [11,46]. 

### 3.3. Chemical Characterization

#### 3.3.1. Quantitative Phytochemical Screening (TPC, TFC, TF, TAC)

The phytochemical dosages were performed using different solvents to determine the concentrations of TPC, TFC, TF, and TAC in AMSD1 seeds. The results are presented in Table 2 The high concentration of TPC and TAC was obtained by ethanol with 395.390 mg EGA/g and 0.534 mg/L, respectively. However, concentrations of 69.247 and 65.052 (mg EQ/g) were obtained for TFC and TF using methanol and diethyl ether as solvents, respectively. 

#### 3.3.2. HPLC-ESI-FULL-MS Analysis

A qualitative chemical screening was conducted using HPLC-ESI-FULL-MS for all extracts (hexane, diethyl ether, acetone, chloroform, ethanol, methanol, and water) of the AMSD1 sample (Table 3). Consistent with previous studies [46,47], our analysis revealed the presence of significant bioactive compounds. The detection of inactive cannabinoids like CBDA and Dihydrocannabinol aligns with their well-known precursor roles in active cannabinoids’ biosynthesis. Additionally, the identification of specific polyphenols, such Cannabisin A, B, and C, in the ethanol extract, agrees with previous research [48,49] and underscores the unique chemical diversity within different extracts. The presence of phosphatidylinositol and tetrasaccharides hydrate, also supports previous reports on the diverse functional compounds present in *Cannabis* seeds [48]. Moreover, the detection of isomers, such as Cannabisin B [isomer 1], echoes findings from other studies emphasizing the intricate chemical complexity of *Cannabis* seeds [50]. Collectively, our results align with and build upon prior research, providing valuable insights into the functional potential of Moroccan *Cannabis sativa L.* seeds.

The most important results are presented below. Other chromatograms and mass spectra are detailed in the Supplementary Material attached to this article.

#### 3.3.3. Specific GC-MS-MS (TQ) Analysis

*Cannabis* seeds are rich in nutritional constituents and non-cannabinoid organic compounds, renowned for their bioavailability and bioreactivity. A qualitative analysis was performed on the seven extracts obtained from the AMSD1 sample, and the resulting chromatograms were processed and compiled (see attached sup. info.). This comprehensive analysis revealed the presence of over 60 compounds, with the most significant 24 compounds listed in Table 4. These volatile components were semi-quantitatively identified by comparing their characteristic mass fragmentation patterns with entries in the Wiley Registry 11th Edition/NIST 2017 Mass Spectral Library database (see match factor in Table 3). Notably, this GC-MS-MS (TQ) analysis unveiled seven molecules that were identified for the first time in *Cannabis* seed: 4,5,7-tris(1,1-dimethylethyl)-3,4-dihydro-1,4-epoxynaphthalene-1(2*H*)-methanol, oleamide, 2-palmitoylglycerol, benzenepropanoic acid, Lowinox 242, 2,4-DBAL, and 2,4-di-tert-butylphenol. The composition of these extracts is characterized by a balanced presence of essential nutritional elements, such as fatty acids and fatty nitriles, as well as antioxidant elements (Table 4). These compounds exhibit various properties, including antioxidant, anti-inflammatory, antimicrobial, and potential health benefits. The presence of these compounds underscores the potential of *Cannabis* seeds as a valuable functional food source. 

### 3.4. Biological Activities

Evaluating the biological activities of *Cannabis* seeds is about discovering their potential health benefits and as a functional food. The antioxidant activity evaluated by the 2,2-diphenyl-1-picrylhydrazyl (DPPH) (IC_50_ values), ferric-reducing ability of plasma (FRAP) (EC_50_ values) and total antioxidant capacity (TAC) (µg EAA/mg values) assays for the different AMSD1 *Cannabis* seed extracts and for the controls (Butylated hydroxytoluene (BHT), Quercetin and Ascorbic Acid), are displayed in Table 4. The data showed that each extract exhibited an interesting antioxidant potency, particularly the ethanolic and methanolic extracts with IC_50_ values of 31.63 ± 0.53 µg/mL and 20.28 ± 3.25 µg/mL, respectively, for free radical scavenging activity (DPPH). Acetone, chloroform, diethyl ether, and ethanol extracts showed good antioxidant activity with EC_50_ values of 159.5 ± 13.44 µg/mL, 165.99 ± 14.69 µg/mL, 236.63 ± 5.13 µg/mL, and 300.61 ± 14.69 µg/mL, respectively. For the total antioxidant capacity (TAC) test, the ethanolic extract showed better results (300.694 ± 92.68 µg EAA/mg values). Nevertheless, these activities were almost the same than that found for the synthetic antioxidant BHT, Quercetin, and ascorbic acid (Table 4). This antioxidant effectiveness of our extracts may be attributed primarily to the presence of the new detected compounds such as benzenepropanoic acid and 2,4-DBAL in the volatile fraction and the Cannabisin A, B, and C in the non-volatile part of studied *Cannabis* seeds. 

Concerning the assessing of the antibacterial and antifungal activity, unfortunately, none of the seven *Cannabis* seed extracts exhibited an inhibitory effect on the tested bacterial and fungal strains (see attached sup. info.). Surprisingly, these extracts were found to have a stimulatory effect on bacterial growth during the entire 24 h period of spectrophotometer monitoring. This unexpected result suggests that the *Cannabis* seed extracts may contain compounds that promote bacterial growth rather than inhibiting it. Further investigation is necessary to understand the mechanisms behind this stimulatory effect and to explore the potential implications. For the preliminary cytotoxicity test on the Vero cell line, the obtained results (Table 4) show that the *Cannabis* seed extracts studied are not cytotoxic to Vero cells [51,52,53]. For most solvents (hexane, diethyl ether, acetone, chloroform, ethanol, and methanol), cytotoxic effects were primarily observed at concentrations equal to or exceeding 200 µg/mL, indicating low cytotoxicity. However, the aqueous extract shows significant cytotoxicity at a lower CC_50_ concentration of 25 µg/mL. Nevertheless, special attention should be given to the aqueous extract, warranting further additional studies for toxicity and safety, especially if intended for incorporation into nutritional and cosmetic formulations. Concerning the antiviral activity (anti-SARS-CoV-2), the obtained results showed that all extracts revealed no significant antiviral effect.

### 3.5. Molecular Docking in the Active Sites

The prediction of protein–ligand interactions can be studied by combining methodologies such as virtual screening and computer-aided design. Currently, molecular docking methods are a widely used tool in selecting powerful molecules as a part of virtual screening of large databases. In this work, conducting docking analyses of the identified phytochemicals from volatile fraction of *Cannabis* seeds against specified targets and their binding affinities for the proteins were evaluated based on the docking scores provided in Table 5a. Regarding their antioxidant activity, 2,4-dimethylbenzaldehyde and 2,4-di-tert-butylphenol demonstrated the strongest affinities for the active sites of NADPH oxidase, with glide scores of −5.928 kcal/mol and −5.886 kcal/mol, respectively. Moreover, 2,4-dimethylbenzaldehyde showed moderate antibacterial activity against beta-ketoacyl-[acyl carrier protein] synthase from *Escherichia coli* and *staphylococcus aureus* nucleoside diphosphate kinase active sites with a glide score of −6.684 kcal/mol and −5.747 kcal/mol. 

The non-volatile fraction of *Cannabis sativa* (L.) seeds revealed notable activity of Cannabisin A, B, and C against NADPH oxidase, exhibiting strong inhibitory potential with glide scores of −9.681, −9.709, and −9.083 kcal/mol, respectively. These findings suggest that these molecules serve as potent NADPH inhibitors, indicating their potential as robust antioxidant compounds. In antibacterial assays, Cannabielsoic acid A displayed strong inhibition against *Escherichia coli* with a glide score of −7.241 kcal/mol, but Cannabisin A exhibited the highest activity anti *S. aureus*. Additionally, in antifungal evaluations against *Aspergillus niger* and *Candida albicans*, Cannabisin B and Cannabinolic acid demonstrated significant efficacy with glide scores of −4.809 kcal/mol and −8.66 kcal/mol, respectively (Table 5a).

In Table 5b,c, a comprehensive breakdown is provided regarding the quantity and nature of bonds established between the volatile compounds originating from *Cannabis sativa* (L.) seeds and the respective active sites. To illustrate, 2,4-dimethylbenzaldehyde forged a singular hydrogen bond with residue ALA 300 within the NADPH oxidase’s active site, while simultaneously forming dual hydrogen bonds with residues THR 300 and THR 302 in the active site of *Escherichia coli*’s beta-ketoacyl-[acyl carrier protein] synthase. Additionally, a hydrogen bond was created with the ASN 112 residue in the active site of *S aureus* nucleoside diphosphate kinase. Similarly, 2,4-di-tert-butylphenol established a lone hydrogen bond with residue SER 378 and a Pi–Pi stacking bond with residue TYR118 in the active sites of sterol 14-alpha demethylase (CYP51) from *Candida albicans*.

The 2D and 3D visual representations (Table 5d,e) of the interaction between Cannabisin B and the active site of NADPH oxidase indicated the formation of five hydrogen bonds with residues THR 112, LYS 134, GLU 163, and PHE 425, in addition to a solitary Pi-Pi stacking bond with TYR 159. Cannabielsoic acid A displayed dual hydrogen bonds with residues VAL 304 and HIE 333 in the active site of *Escherichia coli*’s beta-ketoacyl-[acyl carrier protein] synthase. Furthermore, the interaction between Cannabisin A and the active site of *Staphylococcus aureus* nucleoside diphosphate kinase included the formation of five hydrogen bonds with residues GLU 95, ASN 92, GLU 51, LYS 55, and GLY 116, along with a single Pi-Pi stacking bond with HIE 52 and a Pi-cation bond with LYS 55. Additionally, Cannabinolic acid established two hydrogen bonds with residues SER 378 and MET 508, accompanied by two Pi-Pi stacking bonds with residues TYR 118 and PHE 228 in the active site of sterol 14-alpha demethylase (CYP51) from *Candida albicans*. Finally, the interaction between tetrasaccharide hydrate and the active site of *Aspergillus niger*’s beta-1,4-endoglucanase entailed the formation of four hydrogen bonds with residues GLY 234, SER 233, GLU 160, and GLU267.

## 4. Conclusions

To the best of our knowledge, this study is the first research to highlight the genetic polymorphism of the Moroccan *Cannabis sativa* (L.) by optimum DNA extraction from plant seeds through ITS gene sequencing. The genetic profiles of ten seed strains from different regions in the northern Morocco revealed two distinct molecular profiles, namely *Cannabis*, sp. *sativa*, subsp. *sativa*, and *Cannabis*, sp. *sativa*, subsp. *indica*. Furthermore, the genetic material of sample AMSD1 was subjected to sequencing in order to analyze the THCA synthase gene. This analysis resulted in the identification of a novel THCAS sequence documented in the GenBank database with the accession numbers “BankIt2726356 *Cannabis* OR372636” and the submission ID 2726356. Chemical screening using HPLC-ESI-FULL-MS identified 13 non-volatile compounds, including 3 inactive cannabinoids and 3 specific *Cannabis* seeds’ polyphenols. The volatile part of the seed, analyzed by GC-MS-MS, revealed 24 metabolites of significant nutritional and biological importance, including 7 molecules which were discovered for the first time within *Cannabis* seeds. The elemental nutritional composition of the studied Moroccan *Cannabis* seeds revealed a good nutritional quality, with essential minerals, fatty acids, and proteins contributing to their potential health benefits. Antioxidant activity tests showed that the extracts from *Cannabis* seeds, mainly ethanol and methanol, exhibit interesting antioxidant effects. *In silico* analysis provided insights into the binding affinities of identified phytochemicals with selected protein targets, indicating their potential as potent antioxidants and moderate to low antimicrobials. *In vitro* testing did not reveal significant antibacterial activity for any of the extracts. The *Cannabis* seeds can be utilized as alternative food source rich in nutritional compounds and antioxidants, with potential applications in the food, nutricosmetic, and cosmetic industries.

## Figures and Tables

**Table 1:** Nutrient concentration and nutritional % of AMSD1 seeds sample conformed to FDA norms.

Elements	Ca	Cr	Cu	Fe	K	Mg	Mn	Mo	Na	P	Zn
Nutrient concentration (mg/kg)	573.408	<LOD	<LOD	25.328	3755.999	727.008	26.974	<LOD	309.504	2085.695	47.615
Intake	5.791	-	-	0.256	37.936	7.343	0.272	-	3.126	21.066	0.481
Recommended daily intake (FDA)	1300	-	-	18	4700	420	2.3	-	-	1250	11
Nutritional %	0.445	ND	ND	1.421	0.807	1.748	11.846	ND	-	1.685	4.372

**Table 2:** TPC, TFC, TF, and TAC Concentration of AMSD1 by colorimetric dosage and antioxidant activity and cytotoxicity results of seven AMSD1 *Cannabis* seed extracts.

*Cannabis* Seed Extracts										
	AMSD1 Hexane	AMSD1 Diethyl-ether	AMSD1 Acetone	AMSD1 Chlorofom	AMSD1 Ethanol	AMSD1 Methanol	AMSD1 Water			
**Extracts TPC, TFC, TF, and TAC concentration**										
**TPC (mg EGA/g)**	322.750 ± 28.70	358.260 ± 20.96	291.160 ± 15.37	128.410 ± 93.86	395.390 ± 104.88	307.250 ± 89.49	72.470 ± 1.74			
**TFC (mg EQ/g)**	24.004 ± 0.62	65.052 ± 8.73	58.049 ± 2.64	69.809 ± 3.60	54.266 ± 2.02	69.247 ± 2.32	14.753 ± 5.81			
**TF (mg EQ/g)**	16.663 ± 3.50	60.371 ± 0.00	27.899 ± 2.46	53.592 ± 14.44	11.382 ± 0.39	19.996 ± 0.90	0.146 ± 0.68			
**TAC (mg/L)**	0.004 ± 0.07	0.028 ± 0.08	0.150 ± 0.12	0.039 ± 0.08	0.534 ± 0.93	0.055 ± 0.05	0.038 ± 0.45			
**Antioxidant activity (TAC: (µg EAA/mg). DPPH: IC_50_ µg/mL and FRAP: EC_50_ µg/mL)**								**Positive controls**		
								**BHT**	**Quercetine**	**Ascorbic acid**
**TAC**	243.430 ± 3.60 ^a^	235.478 ± 0.45 ^a^	253.611 ± 23.39 ^a^	246.612 ± 9.90 ^a^	300.694 ± 92.68 ^a^	133.677 ± 5.85 ^b^	187.440 ± 82.78 ^b^	691.47 ± 2.81 ^c^	483.63 ± 7.59 ^c^	943.83 ± 10.41 ^d^
**DPPH**	50 ± 12.73 ^a^	33.76 ± 0.35 ^a^	29.82 ± 0.26 ^a^	136 ± 14.14 ^b^	31.63 ± 0.53 ^a^	20.28 ± 3.25 ^a^	209.63 ± 12.05 ^c^	17.68 ± 2.54 ^a^	24.54 ± 2.89 ^a^	29.16 ± 0.96 ^a^
**FRAP**	520.5 ± 13.44 ^b^	236.63 ± 5.13 ^a^	159.5 ± 13.44 ^a^	165.99 ± 7.09 ^a^	300.61 ± 14.69 ^a^	797.12 ± 18.55 ^c^	484 ± 18.38 ^b^	256.44 ± 13.53 ^a^	186 ± 8.49 ^a^	258.45 ± 11.94 ^a^
**Cytotoxicity: CC_50_ (µg/mL)**										
	200	200	200	200	>200	200	25			

The values of the lines with the same letters (a, b, c, and d) do not differ significantly (means ± SD, n = 3, one-way ANOVA; Tukey test, *p* ≤ 0.05). Shapiro–Wilk test (*p* greater than 0.1); *t*-test (*p* less than 0.01).

**Table 3:** Identification of AMSD1 *Cannabis* seed components present in the full spectrum via HPLC-MS-MS (NIST MS Search 2.3 MS-library and MSMS-library) of all AMSD1 extracts.

N°	Proposed Compounds	Rt (min)	MS (*m*/*z*)	MS (Fragments)	Area % per Seeds Extract Type							Match Factor (%)
					AMSD1-Hexane	AMSD1 -Diethyl ether	AMSD1-Acetone	AMSD1-Chlorofom	AMSD1-Ethanol	AMSD1-Methanol	AMSD1-Wather	
**1**	Nd	2.47	658.2	328.9/279.9	-	-	-	-	-	-	3.1	
**2**	Tetrasaccharide hydrate	3.7	683.2	439.1/341.1	-	-	1.26	1.5	10.25	12.35	6.84	60
**3**	Nd	5.8	431.9	334.2/194	-	-	-	1.32	-	-	8.36	
**4**	nd	15	390.2	292.1/194	-	-	3.42	-	-	-	5.74	
**5**	Cannabisin B	17.51	595.4	494.1/396.1/298.1	-	-	15.2	-	18.41	18.36	-	61
**6**	Cannabisin C	21.55	609.1	363.2/265.1	-	-	-	-	2.14	-	-	70
**7**	Cannabisin A	22.77	593.7	409.1/356.1/311.2	-	-	-	-	2.1	-	-	70
**8**	Phosphatidylinositol	26.89	833.6	nd	25.11	21.36	-	-	-	-	-	
**9**	Cannabielsoic Acid A	27.03	373.5	299.2/194.2	-	-	12.65	10.13	-	15.74	-	61
**10**	Cannabinolic Acid,	29.26	353.2	nd	23.05	23.85	18.35	17.25	15.36	10.36	30.84	
**11**	Cannabisin B [isomer 1]	30.44	595.4	395.1/298.1	20.28	26.18	20.13	25.13	-	20.04	25.36	60
**12**	Cannabidiolic acid, CBDA	30.93	357.1	301.2	20.3	19.2	17.85	30.1	20.35	10.09	-	60
**13**	Dihydrocannabinol	32.37	311.1	279.2	10.75	8.12	8.23	7.3	18.14	10.9	-	60

**Table 4:** GC-MS-MS(TQ) chemical composition of AMSD1 seed sample, important compounds, and their biological/nutritional interest (the bold characters in the table mean interesting compounds).

Compund				MS (*m*/*z*)	MS Fragments (*m*/*z*)	Match Factor (%)
Number	Chemical Name	Cas Number	Chemical Classification			
**1**	Phytane	638-36-8	Isoprenoid alkane	282	57, 85, 99, 127, 141, 183, 197, 282	93
**2**	**Benzenepropanoic acid Or Methyl 3-(3,5-di-tert-butyl-4-hydroxyphenyl) propionate**	**6386-38-5**	**Alkylbenzene (class of phenolic esters)**	292	57, 91, 117, 147, 203, 219, 277, 292	69
**3**	**Tris(2,4-di-tert-butylphenyl) phosphite**	**31570-04-4**	**Alkylbenzene (class of phosphites)**	441	57, 91, 147, 191, 237, 308, 426, 441	85
**4**	**2,4-DBAL or 2,4-Dimethylbenzaldehyde**	**15764-16-6**	**Carbonyl (class of aromatic aldehydes)**	134	51,77, 105, 133, 134,	92
**5**	Linoleic acid	56259-07-5	Polyunsaturated fatty acid	265	67, 75, 95, 129, 150, 178, 220, 262, 265	76
**6**	Palmitic Acid	57-10-3	Saturated fatty acid	241	55, 73, 117, 132, 145, 185, 201, 215, 241	87
**7**	Stearic acid	18748-91-9	Saturated fatty acid	269	55, 73, 117, 132, 145, 185, 201, 269	75
**8**	16-Methyl-heptadecanecarboxylic acid	0-00-0	Saturated fatty acid	269	55, 73, 117, 132, 145, 185, 201, 215, 243, 257, 269	72
**9**	Elaidamide	301-02-0	Fatty acid amide	281	59, 75, 112, 126, 154, 184, 192, 222, 238, 264, 281	93
**10**	Palmitamide or Hexadecanamide	629-54-9	Fatty acid amide	255	59, 72, 114, 128, 142, 170, 198, 212, 255	85
**11**	Nonadecanamide	58185-32-3	Fatty amide	297	59, 72, 97, 124, 152, 166, 208, 222, 236, 268, 297	80
**12**	**Oleamide**	**301-02-0**	**Fatty amide**	281	59, 72, 112, 126, 154, 198, 222, 238, 264, 281	91
**13**	Oleionitril	112-91-4	Fatty nitrile	263	55, 83, 97, 122, 150, 164, 206, 220, 234, 263	81
**14**	Monopalmitin	542-44-9	Satureted fatty acid ester: a triglyceride	300	57, 73, 103, 129, 147, 157, 203, 239, 257, 279, 300	61
**15**	7,9-Di-tert-butyl-1-oxaspiro [4.5] deca-6,9-diene-2,8-dione	82304-66-3	Oxaspiro compound	276	57, 91, 109, 135, 161, 175, 205, 217, 233, 261, 276	76
**16**	**4,5,7-Tris(1,1-dimethylethyl)-3,4-dihydro-1,4-epoxynaphthalene-1(2H)-methanol**	**56771-86-9**	**Organic compound**	344	57, 85, 115, 129, 145, 185, 197, 229, 241, 260, 301, 316, 329, 344	87
**17**	2-Palmitoylglycerol	23470-00-0	Organic compound	387	57, 73, 103, 129, 147, 175, 203, 218, 239, 313, 387	79
**18**	3-Trifluoromethylbenzylamine, N, N-dinonyl	0-00-0	Organic compound	427	55, 109, 159, 202, 228, 268, 314, 340, 384, 426, 427	66
**19**	Carbonic acid, monoamide, N-octadecyl-, 2-ethylhexyl ester	0-00-0	Fatty acid amide	425	57, 74, 112, 186, 268, 314, 352, 396, 425	60
**21**	**2,4-Di-tert-butylphenol**	**96-76-4**	**Phenol**	206	57, 74, 107, 135, 163, 175, 191, 206	92
**22**	Ethanamine	2477-39-6	Amine	103	59, 73, 100, 103	93
**23**	5-(2-Methylpropyl) nonane	62185-53-9	Alkyl nonane	184	57, 85, 98, 126, 141	92
**24**	Sucrose	57-50-1	Glycosyl glycoside	222	73, 103, 129, 147, 191, 217, 222	84
**Important compunds** **Structure** **Interest**						
**4,5,7-Tris(1,1-dimethylethyl) -3,4-dihydro-1,4-epoxynaphthalene-1(2H) -methanol (Cas n°: 56771-86-9)**	Oleamide (Cas n°: 301-02-0)	2-Palmitoylglycerol (Cas n°: 23470-00-0)	Benzenepropanoic acid (Cas n°: 6386-38-5)	Lowinox 242 (Cas n°: 31570-04-4)	2,4-DBAL (Cas n°: 15764-16-6)	2,4-Di-tert-butylphenol (Cas n°: 96-76-4)
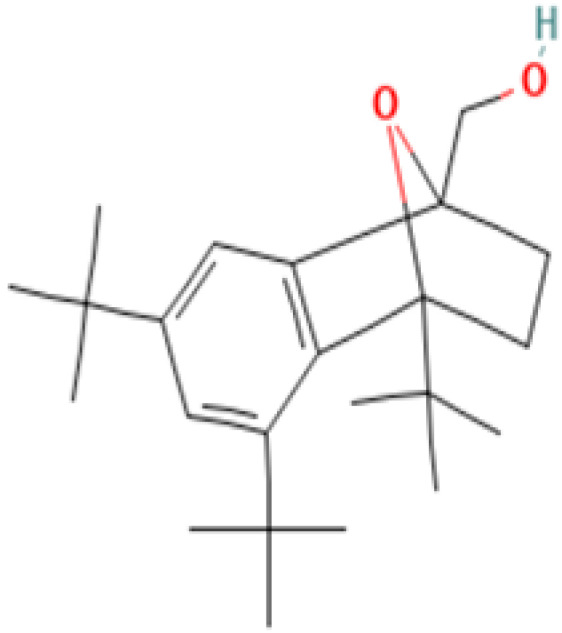	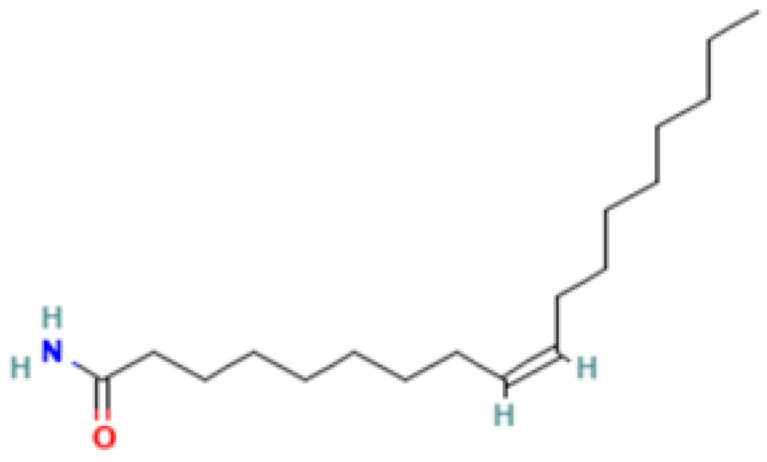	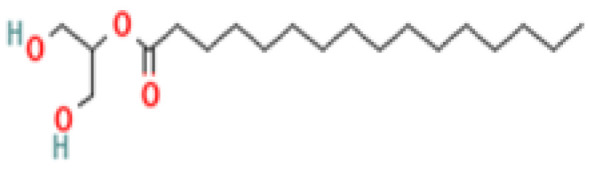	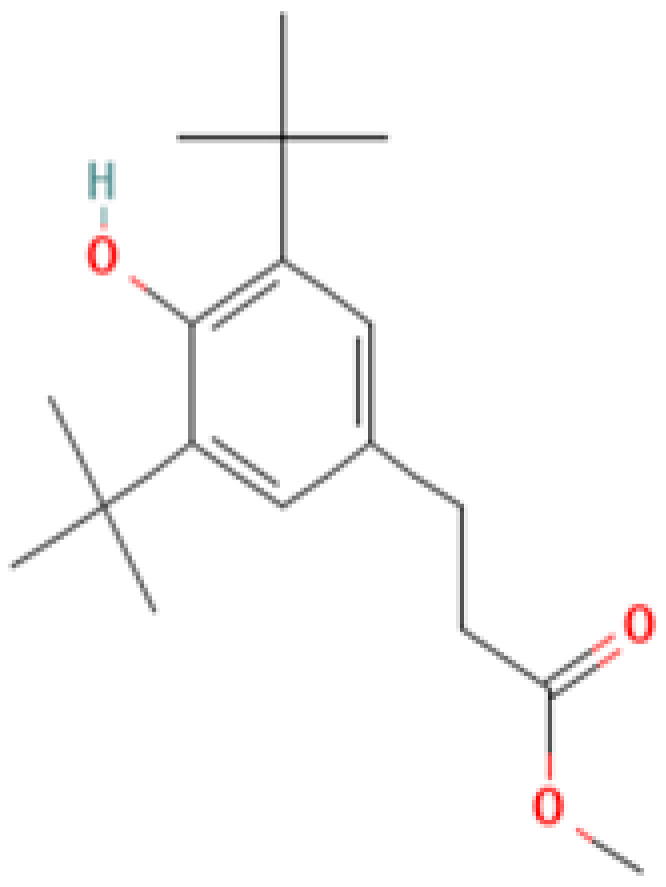	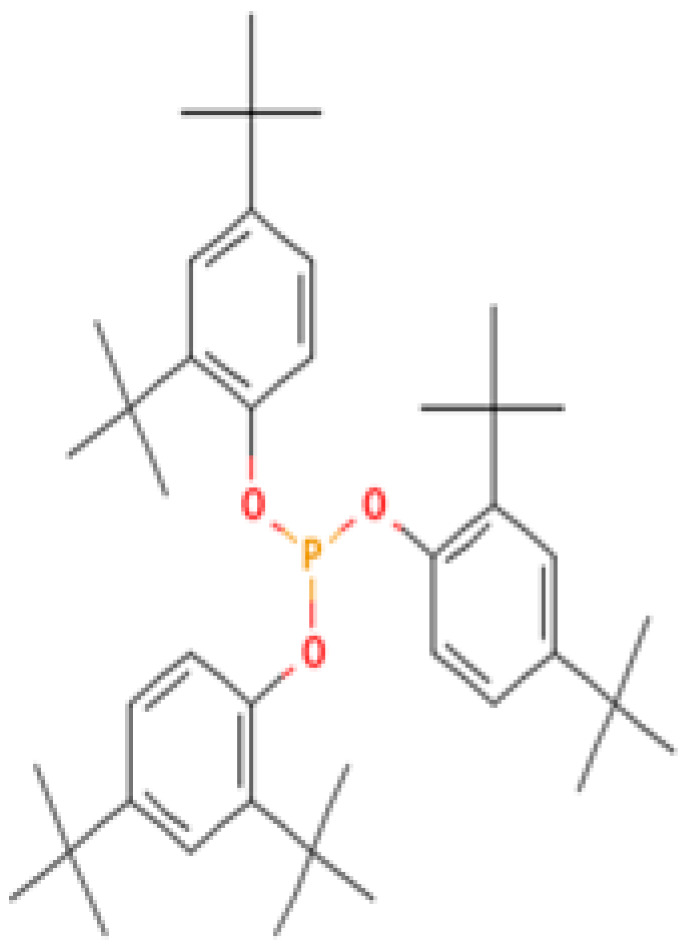	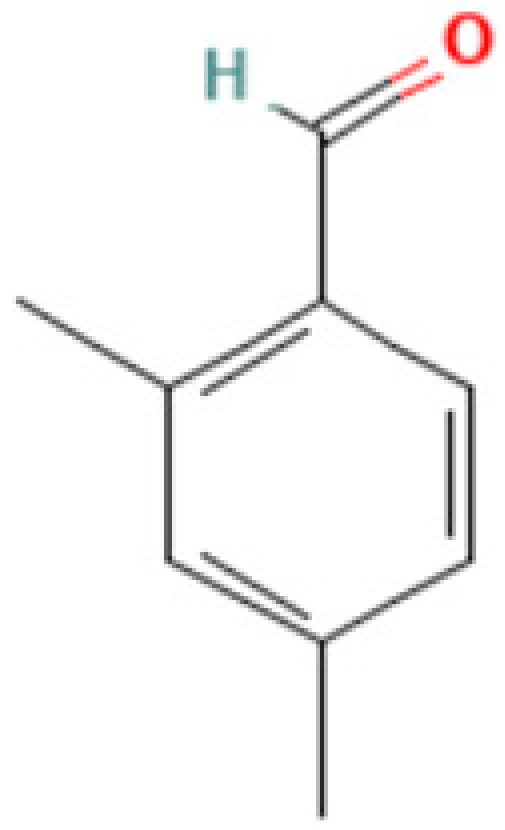	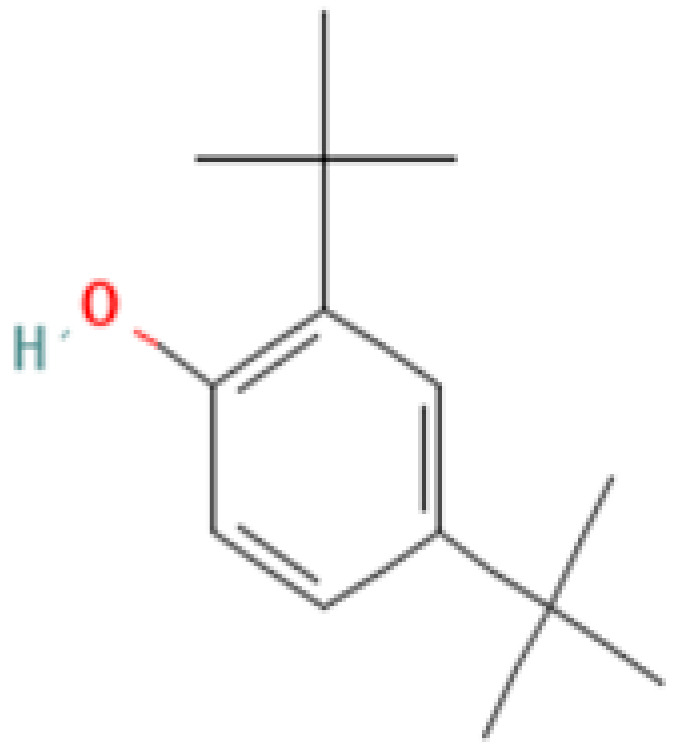
**-Potential health benefits as an antioxidant**	**-Food additive** **-Associated with sleep regulation and anti-inflammatory effects**	**-Ligand for cannabinoid receptors** **-Potential role in inflammation modulation**	**-Antioxydant**	**-Antioxidant properties and preservation of food products**	**Antioxydant** **Food additive**	**Antioxydant**

**Table 5:** Molecular docking analysis results (**a**), the two-dimensional viewer of target ligands in active sites (**b**,**d**), and the three-dimensional viewer of target ligands in active sites (**c**,**e**), for antioxidant and antimicrobial activities evaluation of volatile and non-volatile compounds detected from studied *Cannabis sativa* (L.) seeds extracts.

(a) Docking Results with Ligands in the Active Sites						
***Cannabis sativa (L.)* Seeds Fractions**	**Identified Compounds**	**2CDU**	**1FJ4**	**3Q8U**	**5I77**	**5FSA**
		**Glide Gscore (Kcal/mol)**				
**Volatile compounds detected by GC-MS-MS**	2,4-Dimethylbenzaldehyde	−5.928	−6.684	−5.747	−4.351	−5.319
	2,4-Di-tert-butylphenol	−5.886	−6.386	−3.925	−5.307	−7.964
	2-Palmitoylglycerol	−4.016	−3.646	−3.203	-	−5.496
	3-Trifluoromethylbenzylamine, N,N-dinonyl	−5.813	−3.272	−3.087	−1.687	−7.725
	4,5,7-Tris(1,1-dimethylethyl)-3,4-dihydro-1,4-epoxynaphthalene-1(2H)-methanol	−5.066	-	−4.327	−2.924	−7.733
	5-(2-Methylpropyl)nonane	−3.032	−4.336	−2.904	−2.084	−4.31
	7,9-Di-tert-butyl-1-oxaspiro [4.5]deca-6,9-diene-2,8-dione	−4.986	−4.481	−4.094	−3.834	−6.115
	Carbonic acid, monoamide, N-octadecyl-, 2-ethylhexyl ester	−5.01	-	−2.767	-	−7.149
	Elaidamide	−0.544	−0.968	−0.216	-	−2.635
	Ethanamine	−4.282	−4.658	−3.813	−5.315	−4.856
	Hexadecanamide	−0.088	−1.266	−0.168	-	−2.365
	Linoleic acid	−1.262	−1.59	−1.394	0.975	−2.356
	Methyl 3-(3,5-di-tert-butyl-4-hydroxyphenyl)propionate	−5.298	−4.225	−4.591	−3.651	−7.654
	Monopalmitin	−4.232	−3.247	−3.43	−2.727	−4.874
	Nonadecanamide	−0.451	−0.895	0.328	-	−2.044
	Oleamide	−0.654	−1.771	−0.606	0.183	−2.076
	Oleonitrile	0.411	0.121	-	-	−1.522
	Palmitic Acid	−0.559	−0.627	−0.748	1.384	−1.118
	Phytane	−0.543	−1.562	−0.163	0.405	−2.944
	Stearic acid	−0.212	0.899	−0.79	-	−1.622
	Sucrose	−5.85	−5.077	−4.266	−4.813	−6.103
**Non-volatile compounds detected by HPLC-ESI-FULL-MS**	1-16:0-2-18:2-Phosphatidylinositol	−4.498	-	−4.721	-	−7.728
	7,8-Dihydrocannabinol	−4.576	−5.639	−4.703	−4.184	−8.211
	Cannabidiolic acid	−5.548	−5.63	−5.249	−2.936	−8.029
	Cannabielsoic acid A	−4.821	−7.241	−4.015	−2.994	−6.635
	Cannabinolic acid	−5.49	−5.519	−5.693	−3.331	−8.66
	Cannabisin A	−9.681	-	−6.988	−3.696	−8.273
	Cannabisin-B	−9.709	−5.762	−6.298	−4.809	−8.538
	Cannabisin-C	−9.083	-	−6.271	-	−8.448
	Tetrasaccharide hydrate	−5.303	−5.937	−5.107	−4.769	−6.209
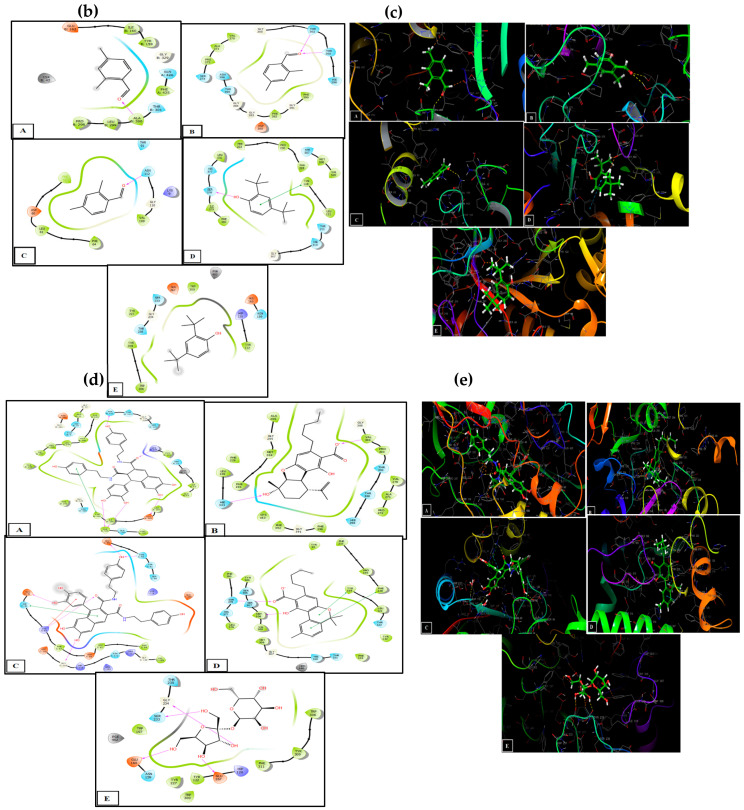						

## Data Availability

Data are contained within the article and supplementary materials.

## Supplementary Materials

The following supporting information can be downloaded at: https://www.mdpi.com/article/10.3390/plants13141938/s1.
