# Supplementary material for: Genetic Characterization and Chemical Identification of Moroccan Cannabis sativa (L.) Seeds: Extraction, and In Vitro and In Silico Biological Evaluation
Source: Plants (Basel). 2024 Jul 15;13(14):1938. doi: 10.3390/plants13141938 (PMC11281279; doi:10.3390/plants13141938)
Supplement: Supplementary file 1 [file plants-13-01938-s001.zip › plants-2805750-supplementary.pdf]

Genetic Characterization and Chemical Identification of Moroccan *Cannabis sativa* (L.) Seeds: Extraction, and *In Vitro* and *In Silico* Biological Evaluation

Amira Metouekel [1], Fadwa Badrana [2], Rabie Kachkoul [3], Mohamed Chebaibi [4], Mohamed Akhazzane [5], Abdelfattah El Moussaoui [6], Nadia Touil [7], Hamid El Amri [2], Elmostafa El Fahime [8,*], Saïd El Kazzouli [1,*] and Nabil El Brahmi [1,*]

Supplementary material

2. Material and methods

2.1. Nutritional quality of cannabis seeds

Table S1: steps of the cannabis seed digestion program for ICP-AES analysis

Digestion program Steps	1	2	3	4	5
**Time (min)**	15	30	15	3	2
**Temperature (°C)**	155	200	50	25	0
**Pressure (bar)**	50	50	50	0	0

2.2. Phytochemical analysis of cannabis seeds extracts

Table S2 : extraction conditions

ORGANIC SOLVENTS	TEMPERATURE (T°)	MACERATION TIME	AGITATION FREQUENCY
Hexane	Room temperature	24 Hours	450 rpm
Ethyl ether	Room temperature	24 Hours	450 rpm
Chloroform	Room temperature	24 Hours	450 rpm
Acetone	Room temperature	24 Hours	450 rpm
Ethanol	Room temperature	24 Hours	450 rpm
Methanol	Room temperature	24 Hours	450 rpm
Distilled water	Room temperature	24 Hours	450 rpm

3. Results and discussion

3.1. Plant identification

3.1.1. Botanical identification

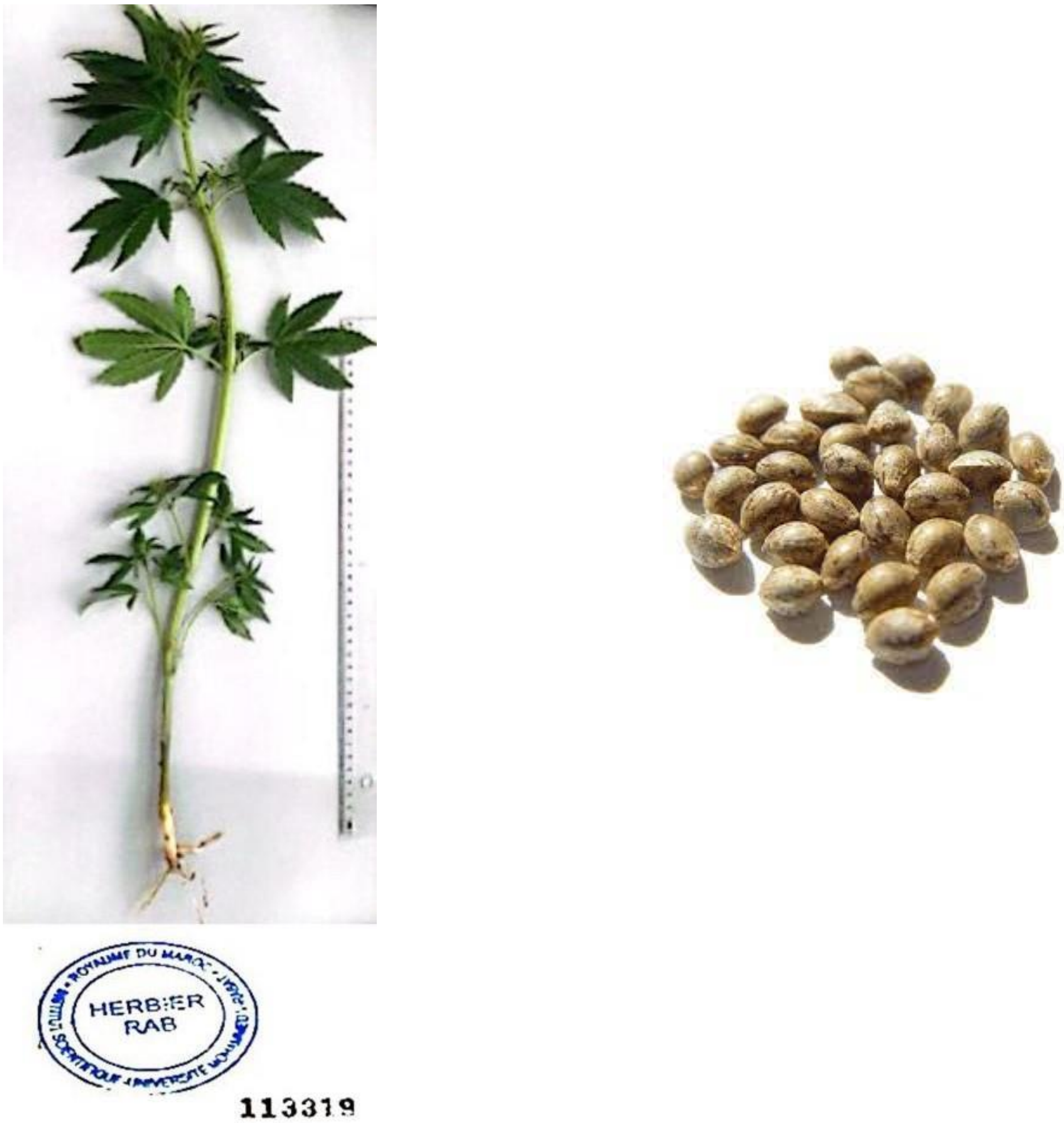

Figure S1: Two-dimensional (2D) scan for AMSD1 cannabis sample botanical identification: Cannabis, sp. sativa: RAB113319

3.1.2. Genetic identification

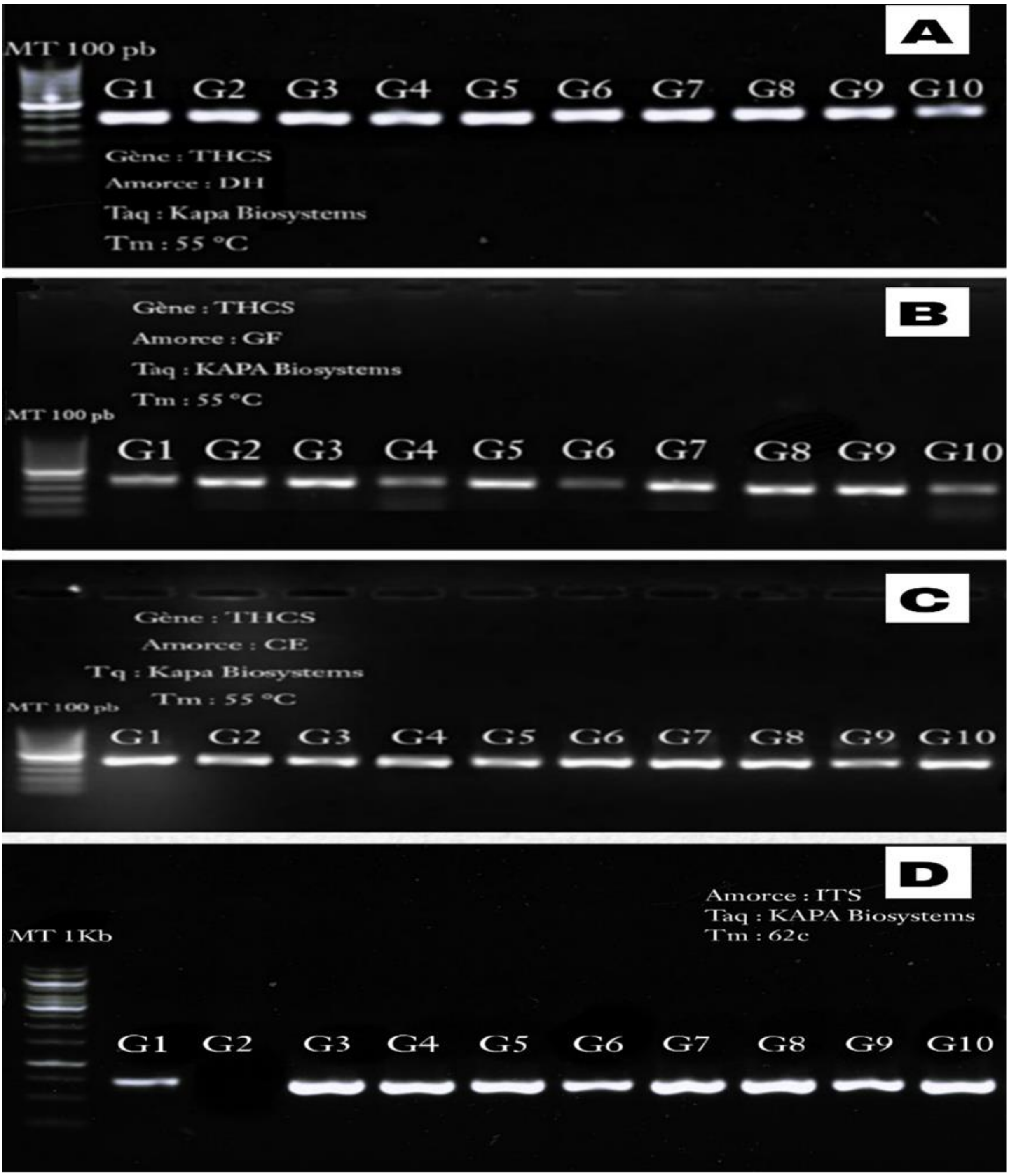

Figure S2 : Electrophoretic migration of PCR products amplified by the primers tested; A: DH(THCAs), B: GF(THCAs), C: CE(THCAs), D: ITS; MT: marker size 100/ 1000bp; G1, G2, G3, G4, G5, G6, G7, G8, G9, G10: the codes assigned for the tested samples.

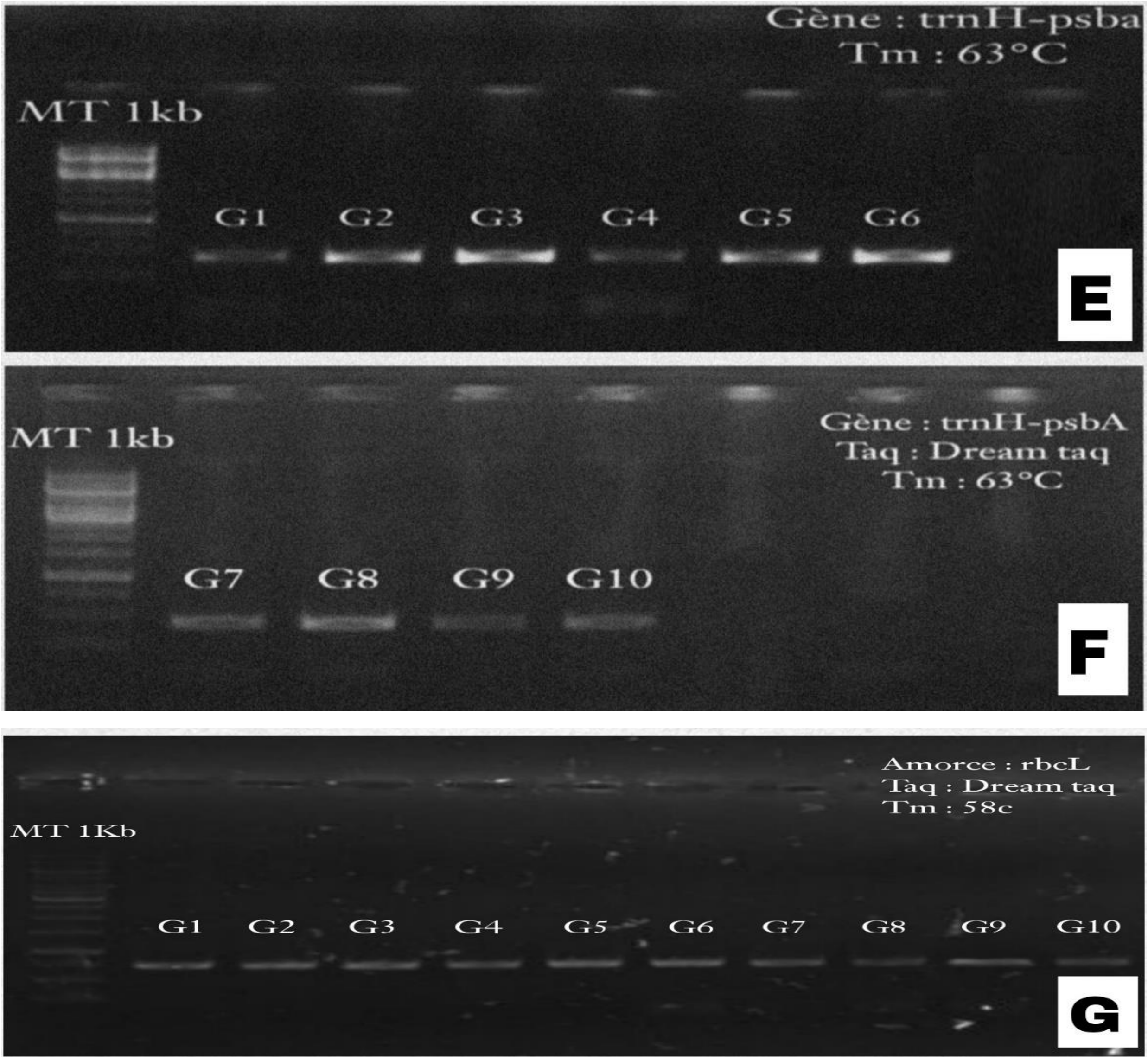

Figure S3 : Electrophoretic migration of PCR products amplified by the primers tested; E and F: trnH-psbA, G: rbcL; MT: marker size 100/ 1000bp; G1, G2, G3, G4, G5, G6, G7, G8, G9, G10: the codes assigned for the tested samples.

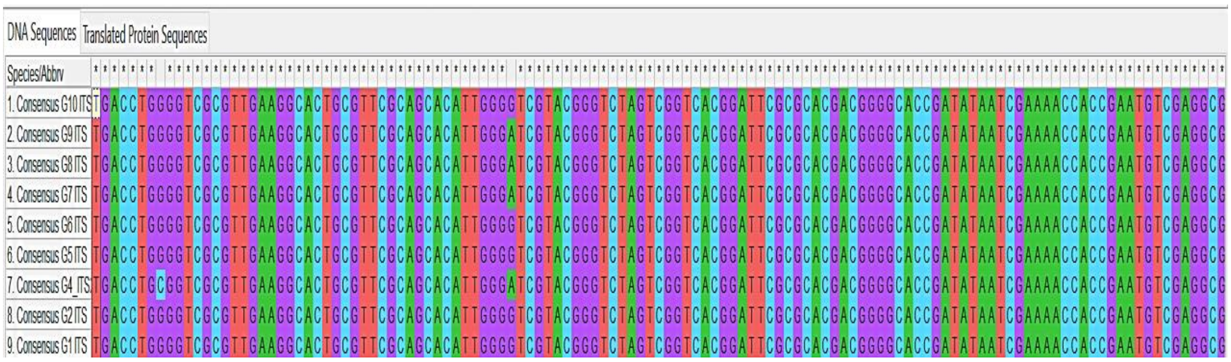

Figure S4 : Multiple alignment portion of ITS gene consensus sequences

3.3.Chemical characterization

3.3.2. HPLC-ESI-FULL-MS analysis

- **HPLC-ESI-FULL-MS Chromatograms for all studied *Cannabis* seeds extracts**

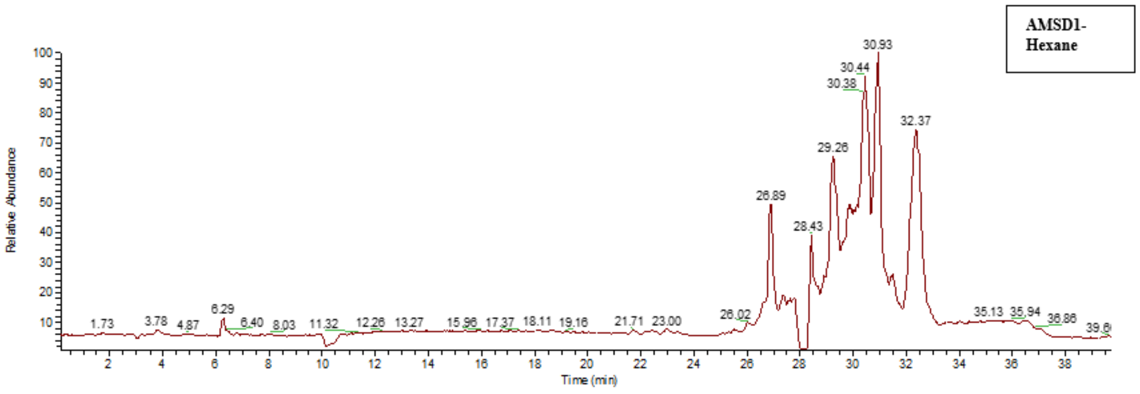

Time (min)

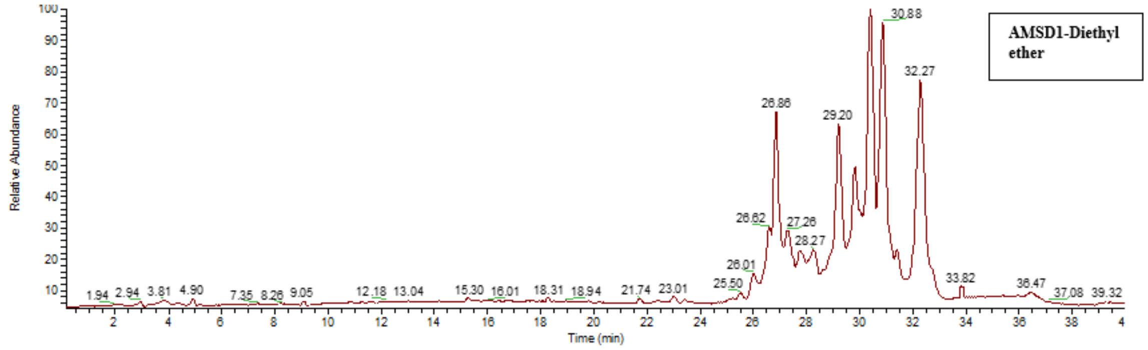

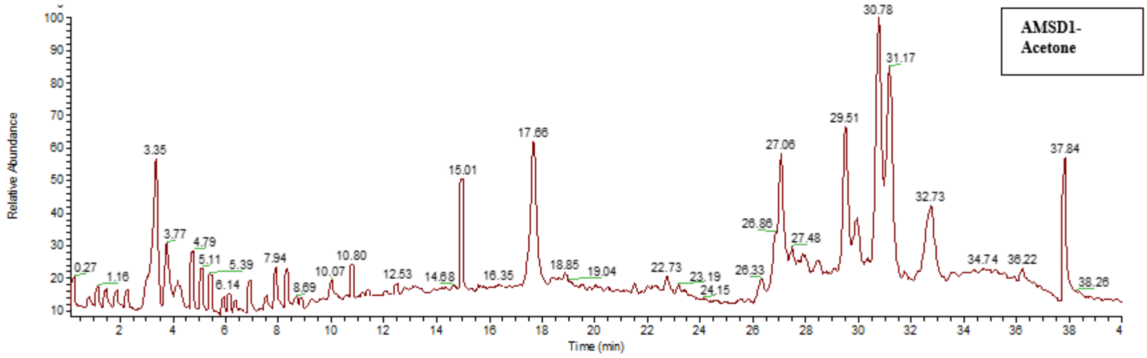

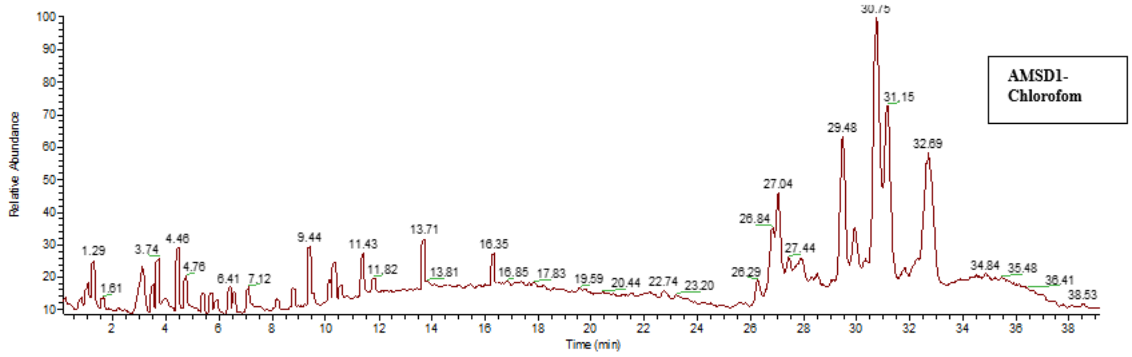

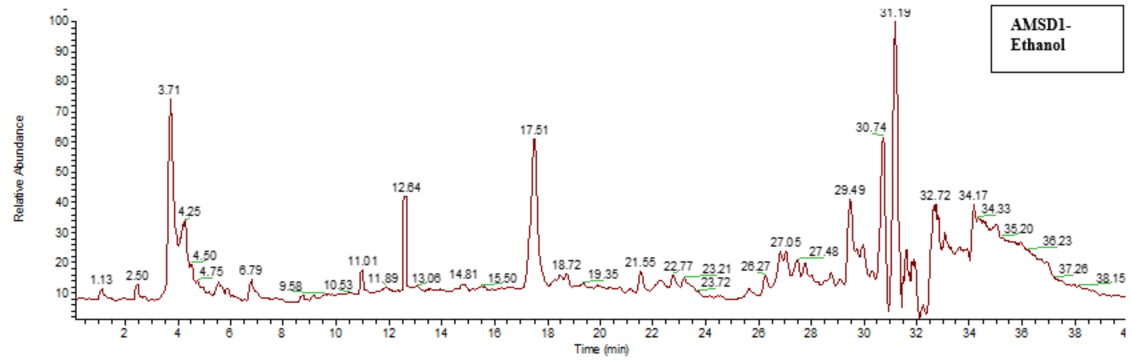

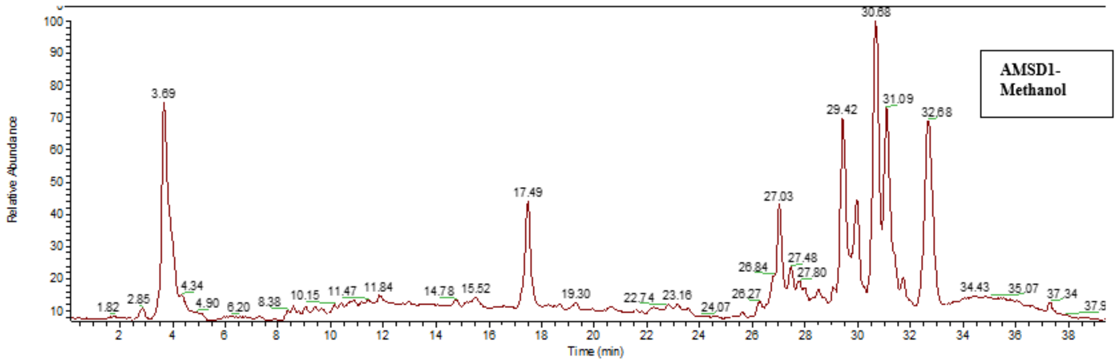

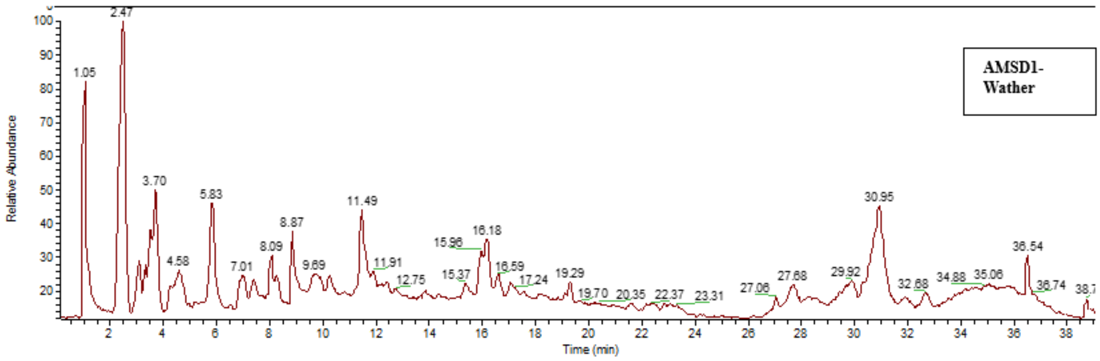

- **Mass spectra for total detected compounds by HPLC-ESI-FULL-MS in all studied *Cannabis* seeds extracts (Nist MS Search 2.3 MS-library and MSMS-library databases)**

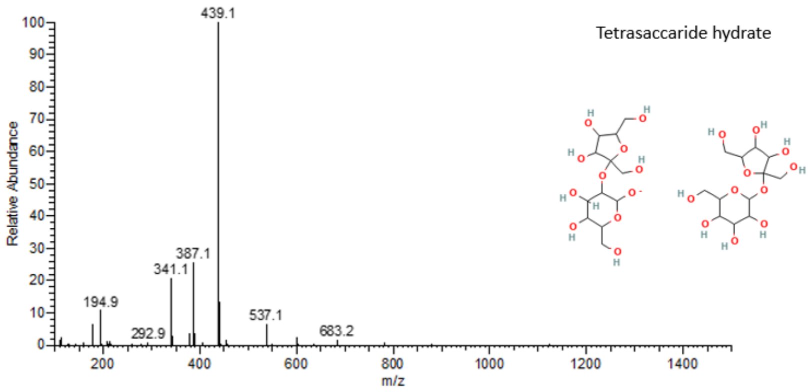

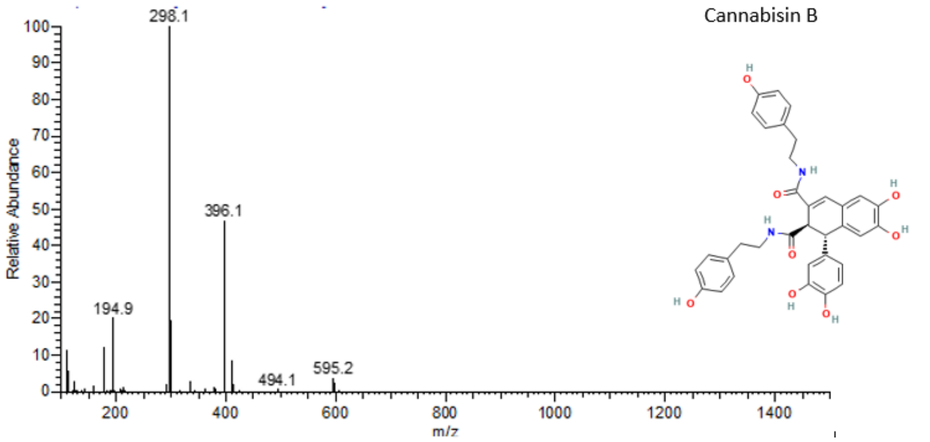
Cannabisin B
Relative Abundance
m/z
298.1
396.1
194.9
494.1
595.2

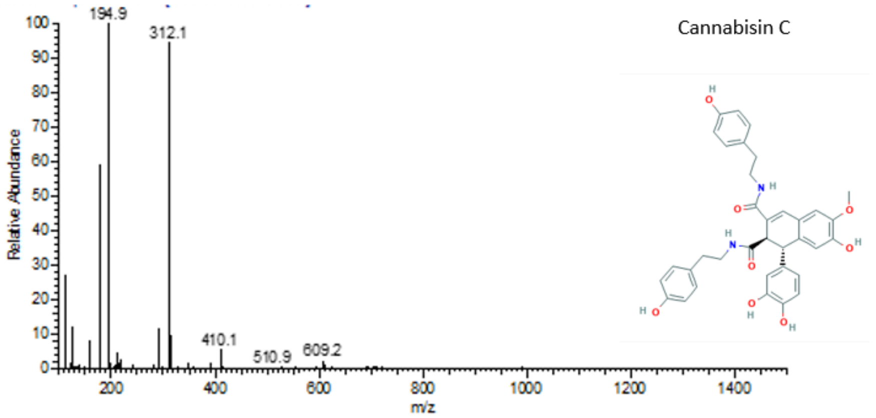
Cannabisin C
Relative Abundance
m/z
194.9
312.1
410.1
510.9
609.2

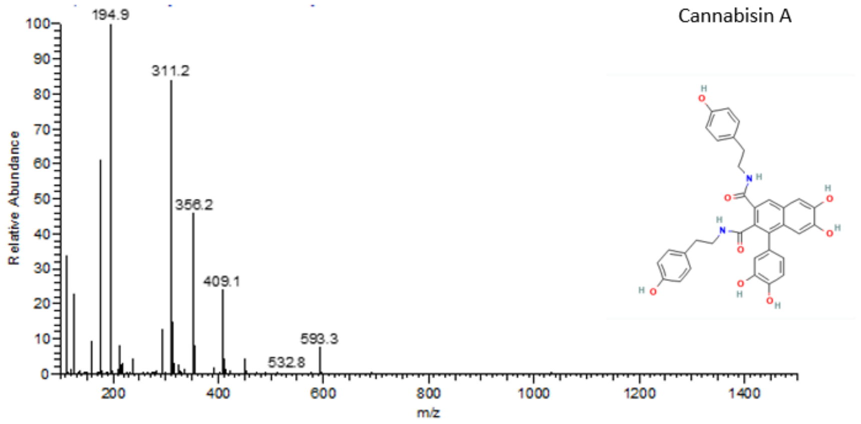
Cannabisin A
Relative Abundance
m/z
194.9
311.2
356.2
409.1
532.8
593.3

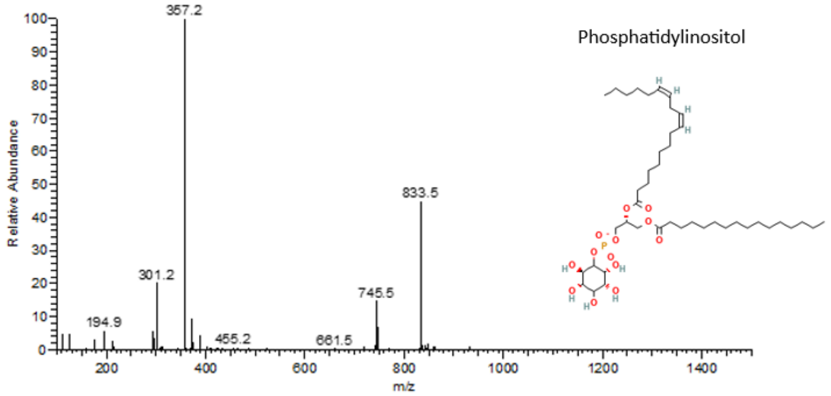
Phosphatidylinositol
Relative Abundance
m/z
357.2
833.5
301.2
745.5
194.9
455.2
661.5

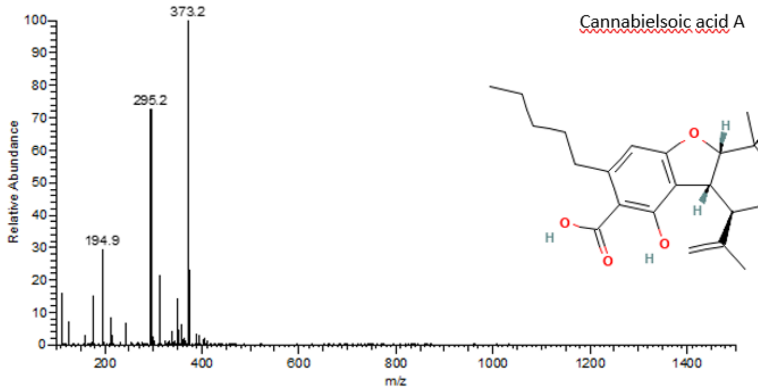
Cannabielsoic acid A
373.2
295.2
194.9
Relative Abundance
m/z

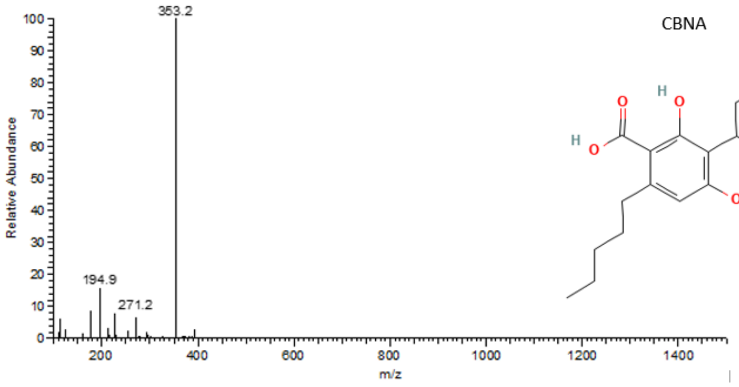
CBNA
353.2
194.9
271.2
Relative Abundance
m/z

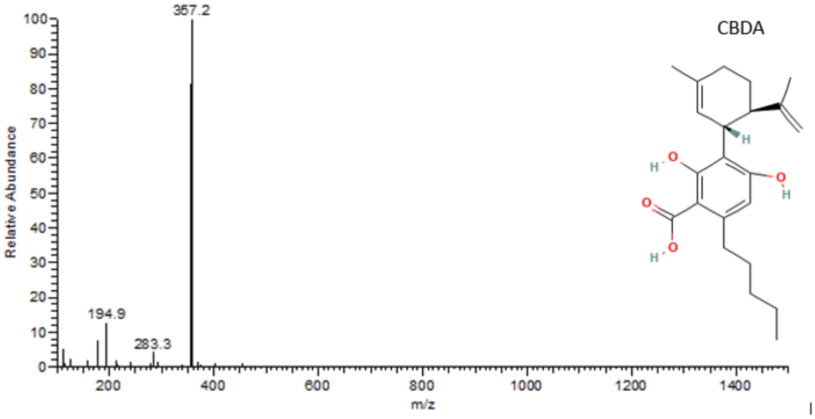
CBDA
357.2
194.9
283.3
Relative Abundance
m/z

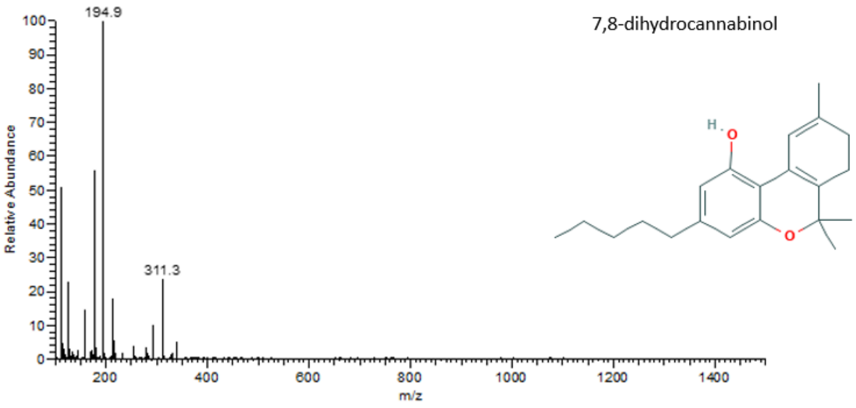
7,8-dihydrocannabinol
194.9
311.3
Relative Abundance
m/z

3.3.3. Specific GC-MS-MS (TQ) analysis

- **Mass spectra by GC-MS-MS (TQ) of the seven volatile compounds detected for the first time in *Cannabis* seed (Wiley Registry 11th Edition / NIST 2017 Mass Spectral Library database)**

CompName:1,4-Epoxynaphthalene-1(2H)-methanol, 4,5,7-tris(1,1-dimethylethyl)-3,4-dihydro-

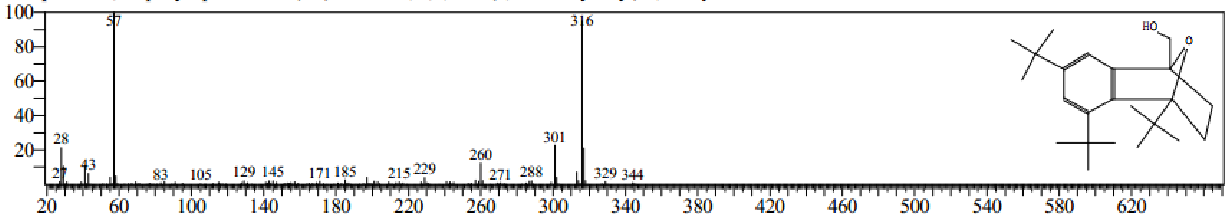

CompName:9-Octadecenamide, (Z)- $$ Adogen 73 $$ Oleamide $$ Oleic acid amide $$ Oleyl amide $$ Slip-eze $$ Armoslip CP $$ Crodamide O $$ Croda

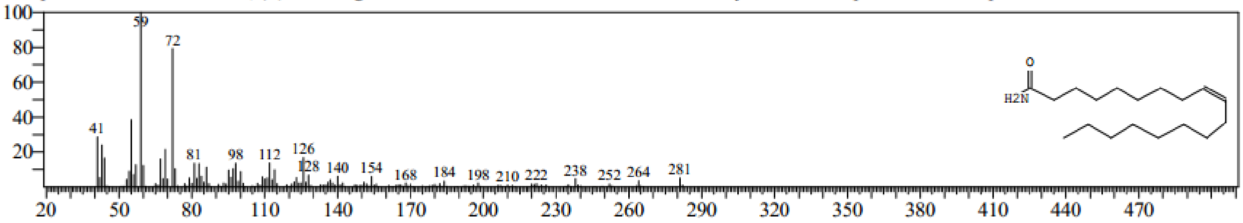

CompName:2-Palmitoylglycerol, 2TMS derivative $$ 2-Monopalmitoylglycerol trimethylsilyl ether $$ Hexadecanoic acid, 2-[(trimethylsilyl)oxy]-1-[[(trime

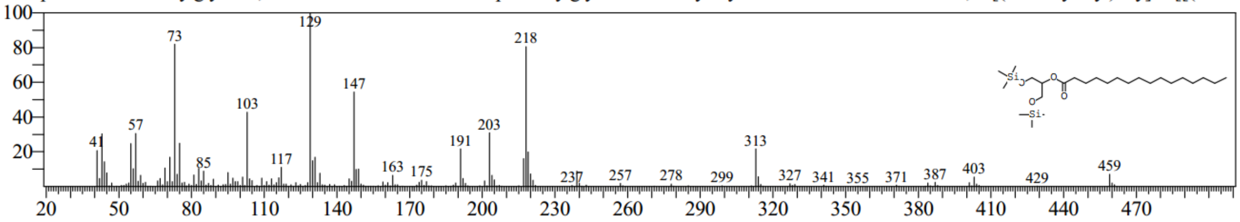

CompName:Benzenepropanoic acid, 3,5-bis(1,1-dimethylethyl)-4-hydroxy-, methyl ester $$ Methyl 3-(3,5-di-tert-butyl-4-hydroxyphenyl)propionate $$ Met

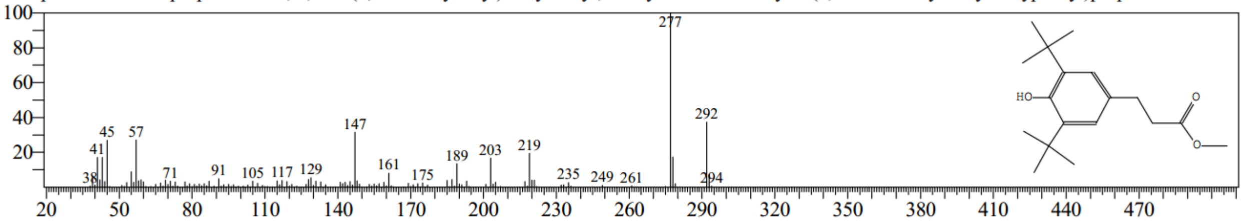

CompName:Phenol, 2,4-bis(1,1-dimethylethyl)-, phosphite (3:1) $$ Alkanox 240 $$ Hostanox PAR 24 $$ Lowinox 242 $$ Naugard 524 $$ Tris-(2,4-di-t-bu

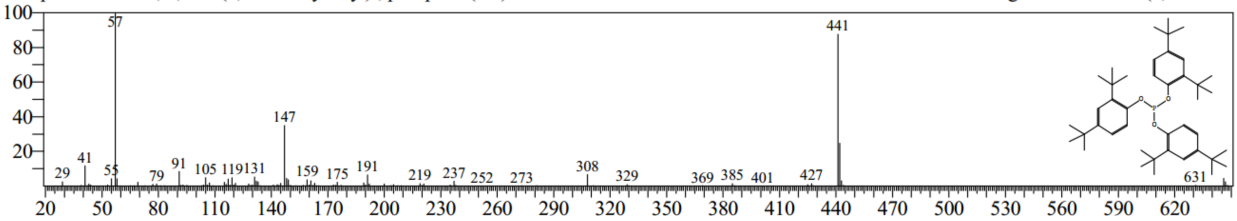

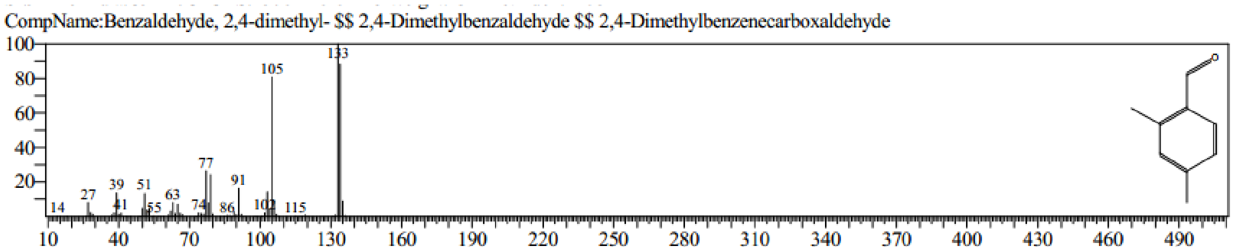

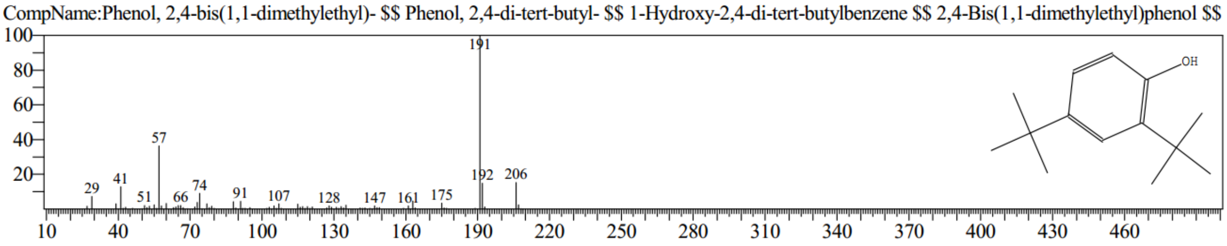

3.4.Biological activities

A- *Escherichia coli* (ATCC B703), gram-negative bacteria: the effect of hexanic extract (similar effect for other extracts) versus the effect of the two positive controls used, Ampicilin and Pinicillin

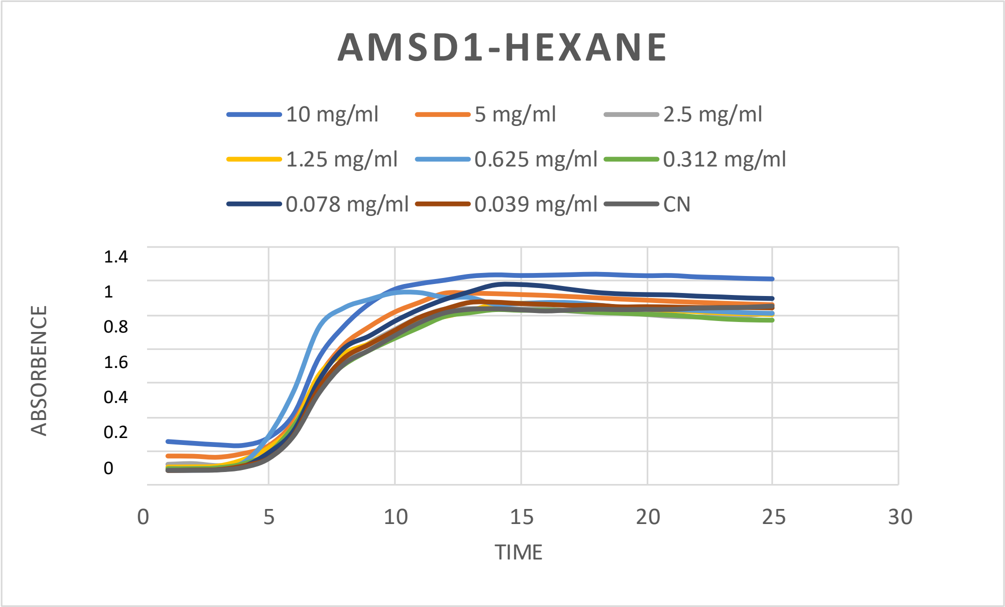

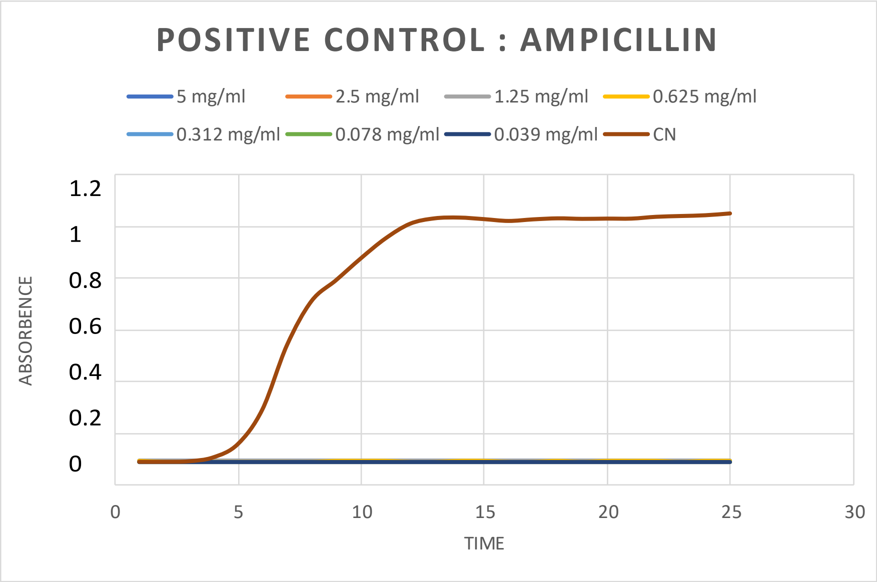

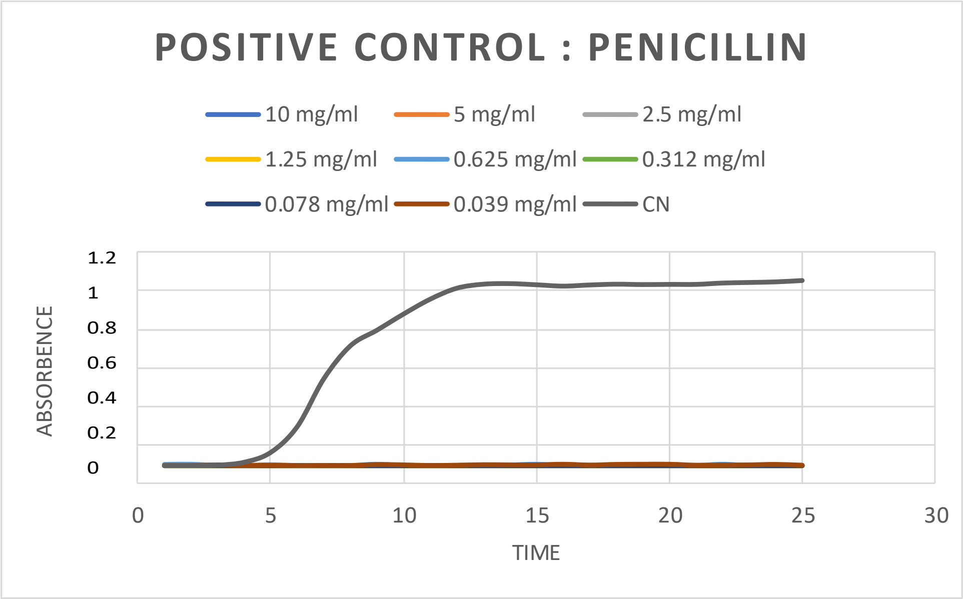

B- ***Staphylococcus aureus*** **(ATCC B803), gram-positive bacteria: the effect of acetone extract (similar effect for other extracts) versus the effect of the two positive controls used, Ampicillin and Penicillin**

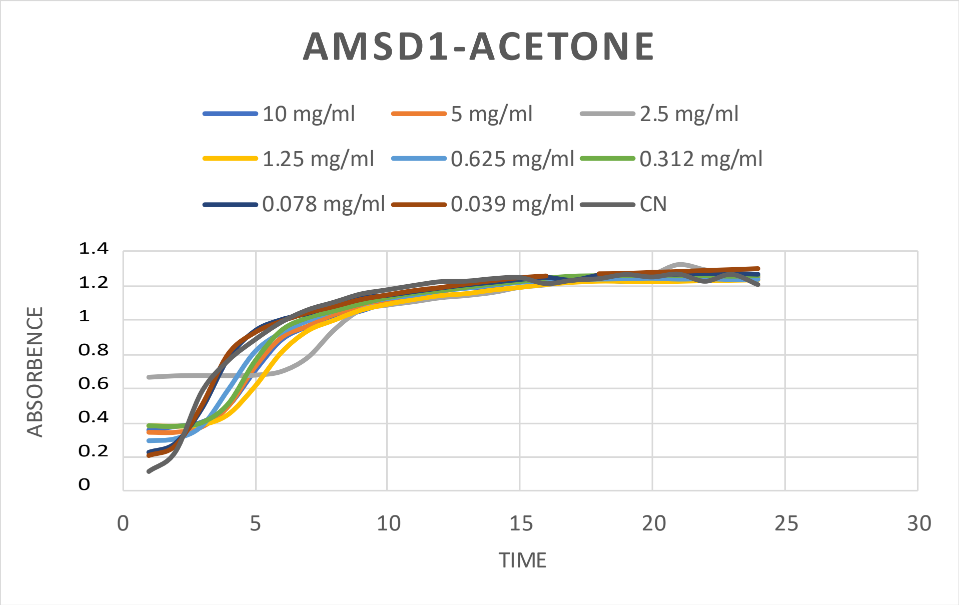
AMSD1-ACETONE
10 mg/ml
5 mg/ml
2.5 mg/ml
1.25 mg/ml
0.625 mg/ml
0.312 mg/ml
0.078 mg/ml
0.039 mg/ml
CN
ABSORBENCE
1.4
1.2
1
0.8
0.6
0.4
0.2
0
0
5
10
15
20
25
30
TIME

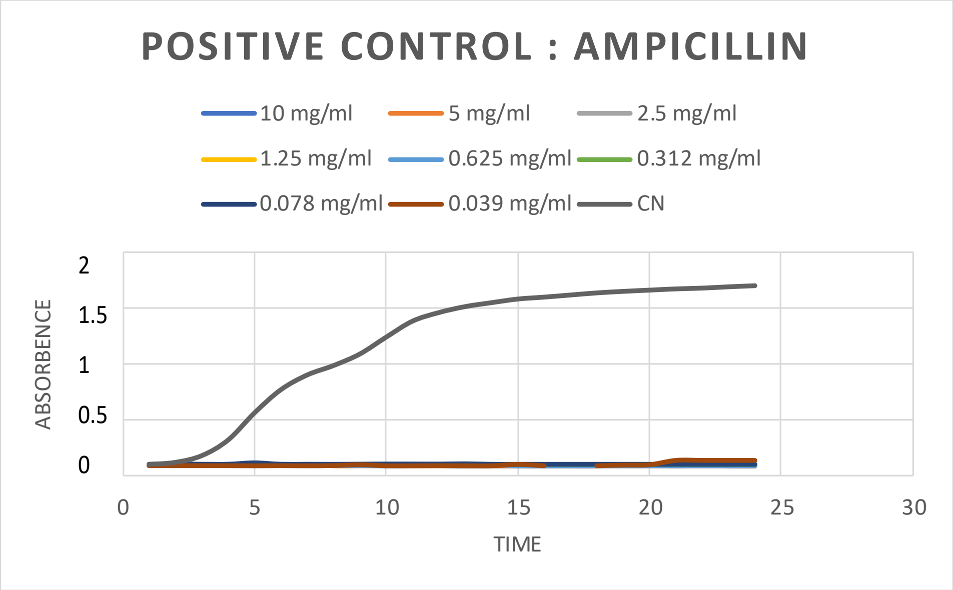
POSITIVE CONTROL : AMPICILLIN
10 mg/ml
5 mg/ml
2.5 mg/ml
1.25 mg/ml
0.625 mg/ml
0.312 mg/ml
0.078 mg/ml
0.039 mg/ml
CN
ABSORBENCE
2
1.5
1
0.5
0
0
5
10
15
20
25
30
TIME

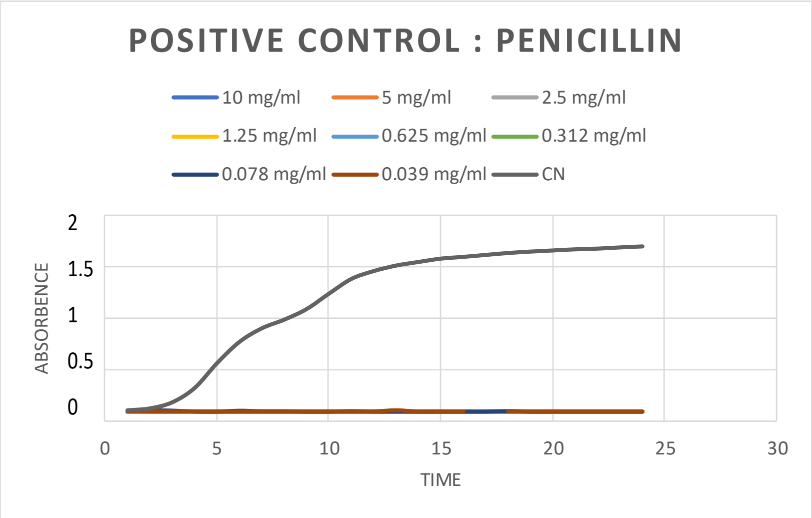
POSITIVE CONTROL : PENICILLIN
10 mg/ml
5 mg/ml
2.5 mg/ml
1.25 mg/ml
0.625 mg/ml
0.312 mg/ml
0.078 mg/ml
0.039 mg/ml
CN
ABSORBENCE
2
1.5
1
0.5
0
0
5
10
15
20
25
30
TIME

C- Disk illustration of the antifungal activity test for the three studied fungi:

***Candida albicans* (IHEM 15856) :**

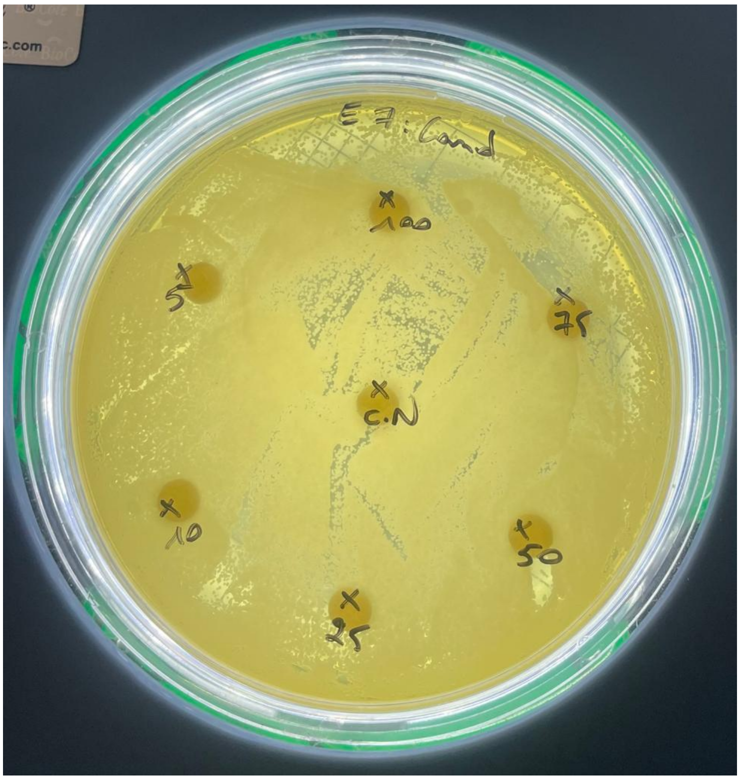

***Aspergillus niger* (IHEM 16879):**

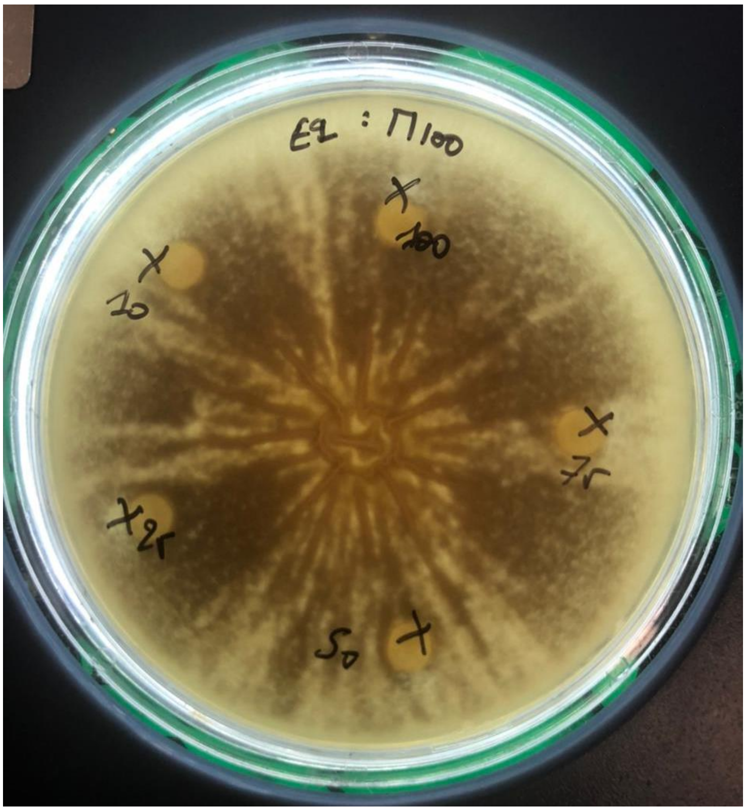

Penicillium crustosum **(MUCL 41820):**

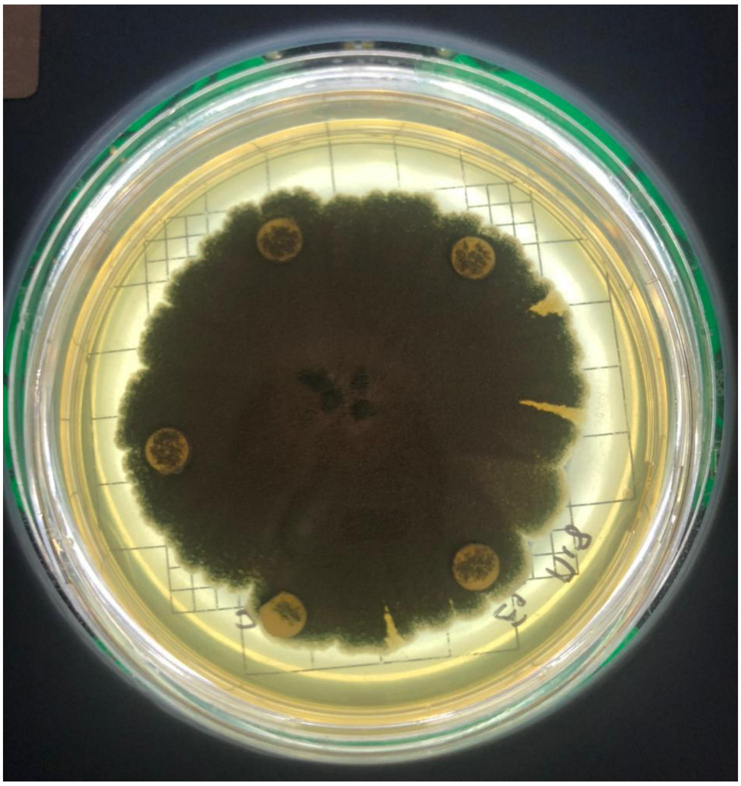

3.5. *In-Silico* and ADMET properties

3.5.2. ADMET analyses

Variant	CNS	Mol MW	SASA	QPlogPo/w	QPlogS	QPlogBB	%HumanOrl Absorption
**2,4-Dimethylbenzaldehyde**	0	134.177	356.402	1.768	-1.682	-0.014	100
**2,4-Di-tert-butylphenol**	1	206.327	467.545	3.853	-3.913	0.128	100
**2-Palmitoylglycerol**	-2	330.507	804.224	4.434	-5.683	-2.287	100
**3-Trifluoromethylbenzylamine, N,N-dinonyl**	2	427.636	904.622	8.893	-8.615	0.652	100
**4,5,7-Tris(1,1-dimethylethyl)-3,4-dihydro-1,4-epoxynaphthalene-1 (2H)-methanol**	0	344.536	619.275	5.427	-5.85	-0.129	100
**5-(2-Methylpropyl)nonane**	2	184.364	532.596	7.509	-7.776	1.259	100
**7,9-Di-tert-butyl-1-oxaspiro[4.5]deca-6,9-diene-2,8-dione**	0	276.375	541.754	2.753	-3.54	-0.192	100
**Carbonic acid, monoamide, N-octadecyl-, 2-ethylhexyl ester**	-2	425.737	935.551	8.828	-8.758	-1.548	100
**Elaidamide**	-2	281.481	730.008	4.35	-4.657	-1.648	100
**Ethanamine**	1	45.084	220.365	-0.131	2.007	0.434	75.914
**Hexadecanamide**	-2	255.443	680.109	3.715	-3.929	-1.546	95.245
**Linoleic acid**	-2	280.45	625.486	5.302	-4.662	-1.304	87.714
**Methyl 3-(3,5-di-tert-butyl-4-hydroxyphenyl)propionate**	0	292,417	620.375	4.577	-5.598	-0.463	100
**Monopalmitin**	-2	330.507	809.289	4.465	-5.773	-2.341	100
**Nonadecanamide**	-2	297.523	767.43	4.844	-5.079	-1.714	100
**Oleamide**	-2	281.481	730.417	4.411	-4.719	-1.565	100
**Oleonitrile**	-2	263.465	709.438	5.953	-7.434	-1.097	100
**Palmitic Acid**	-2	256.428	670.421	5.252	-5.492	-1.46	87.338
**Phytane**	2	282.552	740.498	11.194	-12.094	1.675	100
**Stearic acid**	-2	284.481	730.993	6.065	-6.289	-1.522	93.706
**Sucrose**	-2	342.299	510.841	-3.681	-0.116	-2.7	0
**Tris(2,4-di-tert-butylphenyl) phosphite**	0	646.932	978.535	12.314	-12.071	-0.161	100
**1-16:0-2-18:2-Phosphatidylinositol**	-2	835.063	1430.879	7.159	-7.929	-6.06	31.085
**1-16:0-2-18:2-Phosphatidylinositol**	-2	835.063	1372.156	7.022	-6.932	-5.519	34.055
**7,8-Dihydrocannabinol**	0	312.451	649.597	5.67	-6.757	-0.11	100
**Cannabidiolic acid**	-2	358.477	661.193	5.321	-5.867	-1.216	83.014

**Cannabielsoic acid A**	-2	374.476	672.438	5.14	-5.836	-1.222	82.147
**Cannabinolic acid**	-1	354.445	669.668	5.531	-6.593	-0.983	87.445
**Cannabisin A**	-2	594.62	943.409	2.997	-6.313	-4.718	28.33
**Cannabisin-B**	-2	596.635	873.87	2.073	-4.763	-4.109	21.655
**Cannabisin-C**	-2	610.662	895.151	3.302	-5.201	-3.107	44.184
**Tetrasaccharide hydrate**	-2	342.299	507.688	-3.674	-0.126	-2.616	0.896

MW: The molecular mass falls within 500 atomic mass units.
CNS: The impact on the central nervous system ranges from -2 to +2.
SASA: The solvent-accessible surface area, determined using a probe with a radius of 1.4, falls within the range of 300-1000 radius units.
QPlogPo/w: The predicted partition coefficient between octanol and waterfalls within the range of -2 to 6.5.
QPlogBB: The predicted partition coefficient between blood and brain falls within -3 to 1.2.
QplogS: The predicted aqueous solubility, represented as S in mol/dm³, falls within the range of -6.5 to 0.5.
% HumanOral Absorption: The predicted human oral absorption is expressed on a 0 to 100% scale, where <25% indicates poor absorption and >80% indicates high absorption.
